# Prioritizing Pharmaceutical Contaminants in Great Lakes Tributaries Using Risk‐Based Screening Techniques

**DOI:** 10.1002/etc.5403

**Published:** 2022-07-21

**Authors:** Matthew A. Pronschinske, Steven R. Corsi, Laura A. DeCicco, Edward T. Furlong, Gerald T. Ankley, Brett R. Blackwell, Daniel L. Villeneuve, Peter L. Lenaker, Michelle A. Nott

**Affiliations:** ^1^ Upper Midwest Water Science Center US Geological Survey Madison Wisconsin USA; ^2^ Laboratory & Analytical Services Division US Geological Survey Denver Colorado USA; ^3^ Great Lakes Toxicology and Ecology Division US Environmental Protection Agency Duluth Minnesota USA

**Keywords:** Contaminants of emerging concern, Ecological risk assessment, Ecotoxicology, Pharmaceuticals, Water quality

## Abstract

In a study of 44 diverse sampling sites across 16 Great Lakes tributaries, 110 pharmaceuticals were detected of 257 monitored. The present study evaluated the ecological relevance of detected chemicals and identified heavily impacted areas to help inform resource managers and guide future investigations. Ten pharmaceuticals (caffeine, nicotine, albuterol, sulfamethoxazole, venlafaxine, acetaminophen, carbamazepine, gemfibrozil, metoprolol, and thiabendazole) were distinguished as having the greatest potential for biological effects based on comparison to screening‐level benchmarks derived using information from two biological effects databases, the ECOTOX Knowledgebase and the ToxCast database. Available evidence did not suggest substantial concern for 75% of the monitored pharmaceuticals, including 147 undetected pharmaceuticals and 49 pharmaceuticals with screening‐level alternative benchmarks. However, because of a lack of biological effects information, screening values were not available for 51 detected pharmaceuticals. Samples containing the greatest pharmaceutical concentrations and having the highest detection frequencies were from Lake Erie, southern Lake Michigan, and Lake Huron tributaries. Samples collected during low‐flow periods had higher pharmaceutical concentrations than those collected during increased‐flow periods. The wastewater‐treatment plant effluent content in streams correlated positively with pharmaceutical concentrations. However, deviation from this correlation demonstrated that secondary factors, such as multiple pharmaceutical sources, were likely present at some sites. Further research could investigate high‐priority pharmaceuticals as well as those for which alternative benchmarks could not be developed. *Environ Toxicol Chem* 2022;41:2221–2239. Published 2022. This article is a U.S. Government work and is in the public domain in the USA. *Environmental Toxicology and Chemistry* published by Wiley Periodicals LLC on behalf of SETAC.

## INTRODUCTION

Pharmaceuticals are contaminants of emerging concern because their effects on biota are uncertain, they often lack established water‐quality criteria, and they have been detected in many natural waters throughout the world, including the Laurentian Great Lakes (Blair et al., 2013; Elliott et al., 2017). The presence of pharmaceuticals in water is a result of anthropogenic activity and demonstrates a connection between human and environmental health. Pharmaceuticals have an array of biological targets depending on their intended therapeutic use in humans or domesticated animals and are administered for a wide variety of purposes; however, pharmaceuticals are excreted into the environment and often persist beyond their intended purpose. Active and inactive ingredients can enter the environment from multiple pathways. Wastewater‐treatment plant (WWTP) discharges have been demonstrated to be a significant source in multiple studies (Bartelt‐Hunt et al., 2009; Kosma et al., 2014; Zhou et al., 2009), but pharmaceuticals have also been detected in streams containing no WWTP discharge (Bradley, Journey, et al., 2020), emphasizing the contribution from other sources such as septic systems (Carrara et al., 2008), illicit discharges (Ellis, 2006; Tran et al., 2014), land application of wastewater byproducts (Rogers, 1996), and domestic animal waste (Brown et al., 2006; Sim et al., 2011).

Just as they are designed to impact the health and well‐being of humans and animals, pharmaceuticals have the potential to adversely affect nontarget organisms in the environment. These compounds often target fundamental biological pathways that are conserved across different taxa, thus posing a risk to nontarget organisms (Furuhagen et al., 2014). Behavior, such as cooperation, migration, feeding rates, mating success, parental care, and predator avoidance (Brodin et al., 2014); genetic characteristics; development; reproduction; and even survival of aquatic organisms can be altered by the presence of certain pharmaceuticals at environmentally relevant concentrations (Arnold et al., 2014; Furuhagen et al., 2014; Kolpin et al., 2002). Although the potential adverse effects of pharmaceuticals on nontarget organisms are recognized as a concern, a comprehensive set of water‐quality benchmarks is not available for them. With analytical capability to detect hundreds of pharmaceuticals that lack established water‐quality benchmarks, discerning the chemicals of greatest concern is a challenging task. However, existing resources, such as the US Environmental Protection Agency (USEPA) ECOTOXicology Knowledgebase (hereafter referred to as *ECOTOX*) and data available from the USEPA Toxicity Forecaster (ToxCast) program (Dix et al., 2007; Kavlock et al., 2012) and the interagency Tox21 collaboration (Thomas et al., 2018; Tice et al., 2013; hereafter collectively referred to as *ToxCast*), provide publicly available databases that can aid in the development of screening‐level water‐quality benchmarks to estimate the biological relevance of environmental concentrations.

The ECOTOX database contains in vivo toxicity testing results for thousands of chemicals assembled from primary literature references, including apical effect study results traditionally used to develop aquatic life benchmarks. However, the quantity and type of information are inconsistent among chemicals, and the variable assortment of test organisms, conditions, and endpoints in the database poses challenges when making comparisons of results among chemicals. The ToxCast program uses a standardized set of in vitro, high‐throughput assays to evaluate individual chemicals for interactions with, or effects on, cells, proteins, DNA, RNA, mitochondria, receptors, enzymes, and so on (Judson et al., 2009). In vitro assays are dissimilar to traditional, apical effect studies typically used to develop benchmarks; however, these assays feed into a uniform data analysis routine (Filer, 2019; Filer et al., 2016). This allows for a consistent comparison of relative potency results among tested chemicals and supports the comparative prioritization evaluation sought in the present study (Carpenter et al., 2019; Elliott et al., 2018).

A risk‐based screening approach was used to inform future investigations of pharmaceutical contaminants and resource managers of the largest freshwater ecosystem in North America of the pharmaceuticals and locations deemed to be of the highest relative priority in the present study. Specifically, water samples were collected at 44 diverse tributary locations in variable hydrologic and wastewater effluent conditions and analyzed for a suite of up to 257 pharmaceuticals and nine nonpharmaceuticals. Sample data were used to evaluate the prevalence of pharmaceuticals in Great Lakes tributaries, and the potential biological effects resulting from exposure to the detected pharmaceuticals were assessed using summary metrics from ToxCast and screening‐level benchmarks derived from ECOTOX. All of this information was used to discern which pharmaceuticals are of greatest concern at different locations.

## METHODS

### Site selection and field sampling

**Figure 1:**
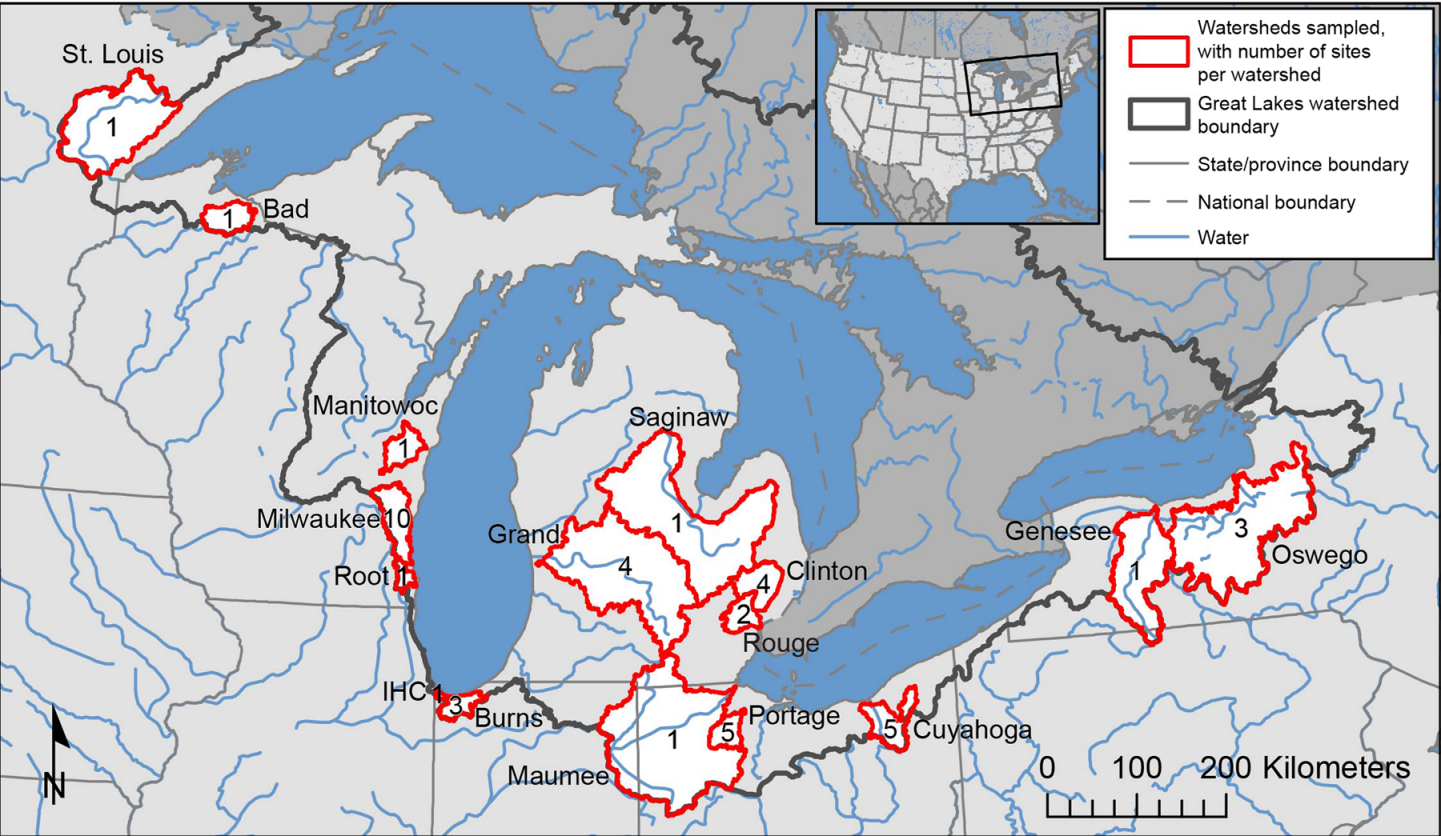
Map of the Great Lakes watershed and the basins sampled within it (labeled). The number of sampling sites used for surface water monitoring in Great Lakes tributaries, October 2017–September 2018, is denoted within each basin. IHC = Indiana Harbor Canal.

Samples were collected at 44 sites within 16 watersheds on tributaries that included two sites draining to Lake Superior, 20 to Lake Michigan, one to Lake Huron, 17 to Lake Erie, and four to Lake Ontario (Figure 1 and Table 1; Supporting Information, Table SI‐1). Sampling sites were selected to characterize Great Lakes watersheds with diverse land‐cover characteristics and a wide range of WWTP effluent contribution amounts. The drainage areas of the tributaries at the sampling points ranged from 22.6 to 16,300 km^2^ (Table 1). Urban land cover ranged from 2.6% to 98.7% in the selected watersheds, and agricultural land cover ranged from 0.2% to 86.1% (Jin et al., 2019; Supporting Information, Table SI‐1). Annual WWTP contributions to streams varied from 0% to 44.1% of streamflow, and population densities ranged from 2.8 to 2260 people/km^2^ within the selected watersheds (Table 1). Eight of the sampled watersheds were monitored at more than one location to allow for comparison of pharmaceutical compound prevalence in different areas of the watersheds with variable WWTP effluent discharges and urban land cover. These sites included three watersheds in the southern Lake Michigan drainage area (Milwaukee River, Burns Waterway, and Grand River), two watersheds in the Lake St. Claire–Detroit River corridor (Clinton River and River Rouge), two watersheds in the western Lake Erie drainage area (Portage River and Cuyahoga River), and one watershed in the Lake Ontario drainage area (Oswego River; Supporting Information, Figures SI‐1–7).

**Table 1:** Watershed characteristics for Great Lakes tributary sites monitored for pharmaceuticals, October 2017–September 2018

Lake	Watershed	Site name	Drainage area (km^2^)	Population density (people/km^2^)	WWTP flow as a fraction of river discharge
Superior	St. Louis	St. Louis	8890	9.17	0.00421
Superior	Bad	Bad	1550	2.79	0.00053
Michigan	Manitowoc	Manitowoc	1340	24.5	0.0113
Michigan	Milwaukee	Milwaukee HWY M	666	80.5	0.0535
Michigan	Milwaukee	Cedarburg	324	109	0.067
Michigan	Milwaukee	Milwaukee	1790	195	0.0456
Michigan	Milwaukee	Milwaukee Walnut	1800	233	0.0456
Michigan	Milwaukee	Menomonee Friestadt	29.5	67.1	0
Michigan	Milwaukee	Underwood	22.6	520	0
Michigan	Milwaukee	Menomonee Church	288	579	0
Michigan	Milwaukee	Menomonee 25th	355	966	0
Michigan	Milwaukee	Kinnickinnic	61.5	2260	0
Michigan	Milwaukee	Milwaukee Mouth	2240	434	0.035
Michigan	Root	Root Clayton	506	334	0.0191
Michigan	IHC	Indiana HC	99.6	914	0
Michigan	Burns	Salt Creek	178	341	0.0929
Michigan	Burns	Burns at 20	429	424	0.0558
Michigan	Burns	Burns	857	345	0.0699
Michigan	Grand	Grand Dimondale	2030	94.3	0.0532
Michigan	Grand	Grand Delta Mills	3290	139	0.0969
Michigan	Grand	Grand Iona	7440	87.9	0.0576
Michigan	Grand	Grand Eastmanville	13,700	109	0.0662
Huron	Saginaw	Saginaw	15,500	69.4	0.0432
Erie	Clinton	Clinton Sterling	811	451	0.076
Erie	Clinton	Red Run	242	1560	0.441
Erie	Clinton	N Br Clinton	472	61	0.0225
Erie	Clinton	Clinton	1900	588	0.103
Erie	Rouge	Rouge	476	965	0
Erie	Rouge	Middle Rouge	283	942	0.072
Erie	Maumee	Maumee	16,300	54.2	0.0611
Erie	Portage	M Br Portage	559	26.3	0.0143
Erie	Portage	S Br Portage	256	36.5	0.151
Erie	Portage	N Br Portage US	102	42.2	0
Erie	Portage	N Br Portage DS	112	39.7	0.297
Erie	Portage	Portage	1120	44.1	0.0733
Erie	Cuyahoga	Cuyahoga Old Portage	1050	297	0.0257
Erie	Cuyahoga	Cuyahoga Ira	1240	313	0.208
Erie	Cuyahoga	Tinkers	246	462	0.149
Erie	Cuyahoga	Cuyahoga	1840	326	0.142
Erie	Cuyahoga	Cuyahoga Cleveland	2040	435	0.268
Ontario	Genesee	Genesee	6400	44.6	0.00612
Ontario	Oswego	Seneca Baldwinsville	8120	52.9	0.0251
Ontario	Oswego	Seneca Belgium	8950	81.4	0.0612
Ontario	Oswego	Oswego	13,200	78.7	0.0401

Drainage areas were calculated in a geographic information system, using basins derived from the Watershed Boundary Dataset (US Department of Agriculture‐Natural Resources Conservation Service et al., 2009). Land‐cover statistics were summarized from the 2016 National Land Cover Database (Jin et al., 2019; Wickham et al., 2021; Yang et al., 2018). Basin population densities were calculated using 2010 US Census data (US Census Bureau Geography Division, 2010). Wastewater‐treatment plant (WWTP) flow as a fraction of river discharge was calculated using WWTP effluent data from the International Joint Commission (Laitta, 2016) and the US Geological Survey SPARROW program (Maupin & Ivahnenko, 2011) from 2012 divided by the 2012 annual mean of mean daily discharge values from the National Water Information System; methods were consistent with Baldwin et al. (2016).

HWY = highway; IHC = Indiana Harbor Canal; HC = Harbor Canal; N Br = North Branch; M Br = Middle Branch; S Br = South Branch; US = upstream; DS = downstream.

Sampling sites were categorized into two tiers for this sampling effort. Tier 1 was comprised of 16 sites located in major tributaries to the Great Lakes (Figure 1). Water samples were collected quarterly at each of these sites, and sampling dates were targeted to occur during November 2017, February 2018, April 2018, and July 2018. Tier 2 included 28 additional sites (Supporting Information, Figures SI‐1–7). Two water samples (one during a low‐flow period, one during an increased‐flow period) were collected at all sites in coordination with the final two quarterly sampling events for Tier 1 sites (April and July 2018).

Sample collection methods are described in detail in the Supporting Information. Briefly, composite water samples were collected from lateral stream transects using equal‐width increment methods (US Geological Survey [USGS], 2006). Field duplicate and blank samples were collected concurrently with a subset of regular samples to ensure the quality of water chemistry data. To prevent sample contamination, Teflon equipment was used and properly cleaned prior to the collection of each sample (USGS, 2004).

### Laboratory analysis

Samples collected during the present study were analyzed for pharmaceuticals and other chemicals using two methods, each covering a distinct set of analytes. Both methods are suitable for filtered water samples. The first method, Method 1 (Furlong et al., 2014), was used for all collected samples and included 109 analytes, 106 of which are pharmaceuticals (Supporting Information, Table SI‐2). A summary of this method is provided in the Supporting Information, with full details provided in Furlong et al. (2014). Method reporting limits (MRLs; determined in reagent water) for individual chemicals ranged between 2 and 270 ng/L during the period these samples were analyzed. The USGS's National Water Quality Laboratory annually assesses MRLs using the procedure of ASTM International (2016), as documented in Williams et al. (2015). The median MRL for all analytes was 30 ng/L. Method detection limits (MDLs) were calculated using the procedure of the USEPA (2005), and the majority of MDLs for Method 1, as defined by the 25th and 75th percentiles of MDL distribution, fell between 13 and 80 ng/L.

The second method, Method 2 (Pronschinske et al., 2022), to determine pharmaceuticals in the present study complements and expands the number of chemicals beyond Method 1, including 152 pharmaceuticals and six nonpharmaceuticals (Furlong et al., 2014). This method is functionally similar to the first in that it uses direct aqueous injection of a filtered water sample, separates the chemicals using high‐performance liquid chromatography (HPLC) directly coupled to a triple quadrupole mass spectrometer, and employs multiple reaction monitoring to produce two unique precursor–product ion pairs for identification and quantitation of individual chemicals. However, there are several important differences between the methods. 1) Two separate instrumental analyses are used for Method 2. One analysis uses positive electrospray ionization (ESI), similar to Method 1 (Furlong et al., 2014), whereas the second analysis uses negative ESI. Method 2 was used to determine 158 chemicals (all but one of which had not been previously determined using Method 1): 49 under negative ESI conditions and 109 under positive ESI conditions. One chemical (lorazepam) is shared between the Method 1 and Method 2 positive ESI analysis. 2) A different reverse‐phase HPLC column is used for both instrumental analyses to achieve separation of the chemicals prior to ionization. 3) Different HPLC mobile phases and gradients are used.

The MDLs and MRLs for Method 2 were calculated using the DQCALC procedure outlined in the ASTM standard (ASTM International, 2016), as documented in Williams et al. (2015), and the USEPA's MDL procedure (USEPA, 2005). Pharmaceutical‐specific MRLs for Method 2 are listed in Supporting Information, Table SI‐3. The median method MRL for all analytes for Method 2 was 20 ng/L, and the majority of MRLs for this method, as defined by the 25th and 75th percentiles of MRL distribution, fell between 20 and 81 ng/L. A more detailed summary of Method 2 is provided in the Supporting Information.

All sites and samples were monitored for the 109 analytes in Method 1. A subset of sites and samples were analyzed for 162 analytes (Method 1 and Method 2 with negative ESI only), and a second subset of these sites and samples was analyzed for 266 chemicals (Method 1, Method 2 with negative ESI, and Method 2 with positive ESI). Not all sites and samples were monitored for Method 2 analytes because programmatic support for Method 2 was removed partway through the present study. To minimize potential bias associated with variable numbers of analytes monitored in each sample, chemical detection frequencies were expressed as percentages of sites monitored. Prior to data analysis, water chemistry data were reviewed and curated. Concentration values below the detection limit were considered zero, and concentration values below the MRL were flagged as estimates (Supporting Information, Table SI‐6). By using a value of zero to represent concentrations below the detection limit, lower bias may have been introduced; however, when computing summary statistics, only values greater than detection levels were used. If interference was observed that did not preclude a quantitation estimate, the value was flagged as an estimate. If interference was more severe, no concentration was reported, and the result included an interference flag. Note that some chemicals were routinely reported as estimated values regardless of concentration because of variation in method performance. Criteria for qualifying concentrations as estimates are provided in Childress et al. (1999).

Overall, 263 of the 266 analytes were not detected in any blank samples. All chemical detections in blank samples were less than the MRL for the respective chemicals. Two chemicals (nicotine and oxycodone) were detected from the 11 blank samples assessed for Method 1 analytes, and one chemical (salicylic acid) was detected from the 10 blank samples assessed for Method 2 analytes (Supporting Information, Table SI‐4). Nicotine was detected in three blank samples; oxycodone and salicylic acid were each detected only once. Because blank sample concentration values were among the lowest values detected in regular samples collected during the present study, no adjustments were made to water chemistry data. However, blank sample detections represent a potential source of uncertainty in concentration values for associated chemicals. There were 20 duplicate samples collected and analyzed for Method 1 analytes and 81 duplicate samples for Method 2. More duplicate samples were collected for the verification of Method 2 analytes because the laboratory analysis methods for these chemicals were in development at the time of sample collection. The median relative percent difference among these comparisons was 23%. Detailed blank and duplicate sample results are in the Supporting Information (Supporting Information, Tables SI‐4 and SI‐5).

### Concentration evaluation

To determine whether pharmaceutical concentrations varied with respect to hydrologic conditions, low‐flow sample concentrations were compared with increased‐flow samples on a site‐by‐site basis. Only Method 1 analytes were used in this comparison because not all samples were analyzed for the chemicals in Method 2, and sites which did not have streamflow data and/or a minimum of two samples were also omitted. The sum of Method 1 analyte concentrations from the sample collected during the lowest flow conditions was compared with the summed concentration of the sample collected during highest flow conditions throughout the present study. Further, to more broadly compare hydrologic variability in samples across sites, low‐ and increased‐flow sample concentrations from each site were normalized by the mean sample concentration at each site. Normalized concentrations were compared using a Welch two‐sample *t* test to assess statistical significance. Concentration values were also used to evaluate the effects of WWTP effluent contributions on the prevalence of pharmaceuticals across sites. Again, for the sake of direct comparability, only Method 1 analytes were used to evaluate the effects of WWTP contributions on pharmaceutical concentrations. Median sample concentrations were regressed against the percentage of streamflow attributable to wastewater using available wastewater effluent data from 2012 (because of the absence of wastewater effluent data during the time of sample collection) and checked for statistical significance using the Pearson correlation coefficient; this is further described in the Supporting Information.

### Alternative benchmarks

A simple measure of concentration does not account for the variability in potency inherent to different chemicals. To give biological context to observed concentration values, established water‐quality benchmarks (e.g., aquatic life benchmarks published by the USEPA) are typically used; however, at the time of the present study, such benchmarks could not be located for the detected pharmaceuticals. In lieu of these benchmarks, ToxCast and ECOTOX were each queried for the detected chemicals, and response and effect data from sets of assays and experiments were collated for each chemical; these collective sets of information are herein referred to as *screening values*. Subsets of screening values were subject to verification and processing procedures to derive activity concentration at cutoff (ACC) values from ToxCast and screening‐level benchmarks from ECOTOX. (Derivation methods are described in detail later in the *Methods* section and within the Supporting Information.) These two collections of discrete values (ACC values and ECOTOX‐derived benchmarks) served as screening‐level alternatives to established water‐quality benchmarks (hereafter referred to as *alternative benchmarks*) for the purposes of the present study, namely comparing the relative potencies of detected chemicals and estimating the potential for each chemical to elicit biological effects. Neither resource provided ecotoxicity information for all monitored chemicals; however, by using the two resources in combination, 68 chemicals were represented.

#### ToxCast

The ToxCast program is facilitated by the USEPA Center for Computational Toxicity and Exposure and has tested more than 9000 chemicals using several hundred high‐throughput screening in vitro assays to characterize biological activities for a range of molecular and biochemical responses that represent more than 300 signaling pathways (Kavlock et al., 2012; Tice et al., 2013). The ToxCast program evaluates a wide variety of biochemical activities using in vitro testing techniques but does not necessarily cover all pathways that are functional and relevant in intact, complex organisms. Many bioactivities measured in ToxCast assays are conserved among species, but others are not. It should be noted that ToxCast assays are primarily derived from vertebrate cell lines with an emphasis on prioritizing chemicals for potential human health effects. Because these assays are not focused on aquatic species (especially invertebrates and plants), they may not be fully representative of responses in aquatic invertebrates and plants.

Still, ToxCast has been used to evaluate surface water quality (Blackwell et al., 2017, 2019; Bradley et al., 2019; Corsi et al., 2019), sediment quality (de Baat et al., 2019), drinking water quality (Bradley, Argos, et al., 2020), and chemical burden in wildlife samples. The use of ToxCast data and selection of ToxCast assays for evaluating potential biological effects followed previously published techniques (Blackwell et al., 2017; Corsi et al., 2019) and are described briefly in our study. The ToxCast data were accessed and processed using the ToxEval R package (DeCicco et al., 2018). Version 3.2 of the ToxCast database (US Environmental Protection Agency, 2017) was queried to retrieve assays of relevance for the present study and estimate relative chemical potencies for the associated biological activities. Assays used in this analysis were chosen from the original list of ToxCast assays based on consideration of data‐quality remarks, examination of chemical dose–assay response curves, redundancy of ToxCast information, the nature of the assays, and assay‐associated reliability/quality for detecting gain or loss of signal. This process reduced the original 717 assays with measurable responses (i.e., *hit calls*) for the detected chemicals to 394 assays considered to be appropriate for use in this analysis; additional details are provided in Supporting Information, Tables SI‐2, SI‐7, and SI‐8.

Several summary metrics are modeled from chemical dose–assay response curves in the ToxCast data analysis pipeline. For the present study, the ACC was chosen for comparison with observed water concentrations, consistent with previous efforts (Alvarez et al., 2021; Blackwell et al., 2017; Corsi et al., 2019; Fay et al., 2018). The ACC is determined as a multiplier of the baseline median absolute deviation of measured activity in the assay that provides an indication of the concentration at which the bioactivity measured first exceeds the baseline response. More thorough descriptions of its derivation are provided elsewhere (Filer et al., 2016; Judson et al., 2009). Using ToxCast data, exposure–activity ratio (EAR) values were calculated as the quotient of observed concentrations and the ACC values from ToxCast assays (Equation 1). In addition, to include all assays of effects relevant to the present study, EAR values were summed for each chemical (Equation 2). Because the number of relevant assays conducted varied by chemical, EAR_Chem_ values may be biased toward chemicals that have been more thoroughly assessed. This bias was evaluated and found to be minimal, especially for the relative prioritization approach employed in the present study (Supporting Information, Figure SI‐8).

(1) $$\text{EAR}=\frac{\text{Measured concentration in sample }(\mu M)}{\text{ACC for chemical }‐\;\text{assay pair}(\mu M)}$$

(2) $${\text{EAR}}_{\text{Chem}}=\sum {\text{EAR}}_{\left[i\right]}\;$$

In Equation 2, *i* represents the assays relevant for each individual chemical.

#### ECOTOX

Toxicological data from ECOTOX were used for the development of screening‐level benchmarks for chemicals detected in the present study (US Environmental Protection Agency, 2020); ECOTOX is specifically focused on ecological effects and in vivo test results that often include apical effects. Apical endpoints can integrate and capture the results of perturbation of a broad diversity of pathways; however, it is generally difficult to diagnose exactly which pathways are perturbed based on apical responses alone. For the chemicals queried, ECOTOX returned records for each unique test and result reported in the knowledgebase (Supporting Information, Table SI‐9). Records included attributes for each test performed (e.g., laboratory/environmental conditions, species, effect, concentration, endpoint), which were used to filter results to those relevant to the present study. For each chemical, any lower outlier endpoint concentration values were investigated by consulting their original literature sources for verification of endpoint relevance and accuracy; any revisions were recorded (Supporting Information, Table SI‐10). Application factors (AFs) were used to account for additional levels of uncertainty and provide conservative estimates of benchmark values (a detailed description of the benchmark derivation process is provided in the Supporting Information).

Benchmarks were developed for each detected chemical by dividing ECOTOX endpoint concentration values by the overall AF assessed for the respective endpoint (Equation 3). Overall AF values ranged from 10 to 200 and were computed as the product of three discrete AF values: AF_Endpoint_, AF_Species_, and AF_Persistence_, similar to the process used in Hull et al. (2015).

(3) $$\text{ECOTOX Benchmark}=\frac{\text{Minimum endpoint subset concentration}\left(\mu g/L\right)}{\left({\text{AF}}_{\text{Endpoint}}\times {\text{AF}}_{\text{Species}}\times {\text{AF}}_{\text{Persistence}}\right)}$$

Based on the ECOTOX endpoint code, endpoints were separated into three benchmark subsets (Supporting Information, Table SI‐11): *No Effect* (AF_Endpoint_ = 10), *Low Effect* (AF_Endpoint_ = 10), and *Acute Effect* (AF_Endpoint_ = 100; Supporting Information, Table SI‐12). Generally, No Effect endpoints were defined by no‐observed‐effect concentration (NOEC)/no‐observed‐effect level or effect concentration (EC)/lethal concentration (LC) ≤ 10 endpoint concentration values; Low Effect endpoints were defined by lowest‐observed‐effect concentration/lowest‐observed‐effect level or EC/LC 10 < *x* < 50 endpoint concentration values; Acute Effect endpoints were generally defined by EC/LC50 endpoint concentration values (Supporting Information, Table SI‐11). Endpoints in the Acute Effect subset were assigned an elevated AF_Endpoint_ value because of the assumption that lower‐level effects may be elicited at concentrations considerably lower than those which provoke acute effects. Next, test species data were summarized for ECOTOX records within each endpoint subset. Endpoint subsets with a minimum of three fish test species, three invertebrate test species, and one plant test species were considered well characterized by test species (AF_Species_ = 1); endpoint subsets not meeting the test species criteria were not considered well characterized (AF_Species_ = 2; Supporting Information, Table SI‐12). Finally, the aquatic persistence of each chemical was estimated using the BIOWIN^TM^ 3 Ultimate Survey Model within Estimation Programs Interface Suite^TM^ (US Environmental Protection Agency, 2012), and biodegradation half‐lives were approximated as described in Aronson et al. (2006). Similar to Hull et al. (2015), chemicals with a half‐life shorter than 8 weeks were not considered persistent (AF_Persistence_ = 1); those with a half‐life longer than 8 weeks were considered persistent (AF_Persistence_ = 5; Supporting Information, Table SI‐12). Persistence AFs were not applied to Acute Effect endpoint subsets under the assumption that even nonpersistent chemicals might elicit effects in the short term. Benchmarks were computed for all endpoint subsets wherever possible, and the minimum benchmark computed for each chemical was used for calculating toxicity quotient (TQ) values (Equation 4). In this way, various endpoints, including studies monitoring acute effects and chronic effects, were used to derive ECOTOX benchmarks for the chemicals in the present study. The variability in studies used for benchmark derivation is a source of uncertainty in relative comparisons of ECOTOX‐derived benchmarks among chemicals and did not allow for the summation of TQ values by chemical as was done for EARs. Instead, TQ values were calculated using the minimum benchmark concentration, which resulted in the maximum TQ value for each observed chemical concentration.

(4) $$\text{TQ }=\;\frac{\text{Measured concentration in sample}(\mu g/L)}{\text{ECOTOX }‐\;\text{derived benchmark for chemical}(\mu g/L)}$$

#### Hazard quotients

To interpret the potential biological relevance of water chemistry data, hazard quotients (i.e., EARs and TQs) were computed to normalize the environmentally observed concentrations to levels with the potential to produce adverse effects. The ToxEval R package (DeCicco et al., 2018) was used for hazard quotient computations as well as further data analysis and visualization. For each sample, EAR and/or TQ values were computed for all analytes represented by a screening‐level ToxCast and/or ECOTOX benchmark. Both EAR and TQ values spanned many orders of magnitude; EAR and TQ values of greater magnitude indicate a higher likelihood for adverse effects. However, these two sets of alternative benchmarks each have strengths and limitations. The means by which they were derived for the present study were similar but distinct. In addition, there are differences in the types of effects targeted by the assays and studies included in ToxCast and ECOTOX. In some cases, traditional animal testing will detect toxicity that ToxCast may miss, because of the lack of an assay suitable for the relevant mode of action. However, in many cases targeted and apical testing with intact animals may miss pathway perturbations that can cause chronic, sublethal impacts on health and ecological fitness. Further, the comprehensiveness of these alternative benchmarks depends on screening‐value data robustness for individual chemicals.

### Pharmaceutical prioritization

A framework was developed to determine the relative priority levels of pharmaceuticals represented by the screening‐level alternative benchmarks and to define a list of high‐priority pharmaceuticals. The pharmaceuticals on the list are not guaranteed to be highly toxic, nor are they the only chemicals with the potential for biological effects; however, the list is intended to provide resource managers with information regarding which pharmaceuticals are most likely to elicit adverse biological effects and to inform future investigators of the relative priority and research available for the detected pharmaceuticals. Additional research in the laboratory and in the field would need to be completed to verify that the observed chemical concentrations truly cause adverse effects.

Both ToxCast and ECOTOX provide distinct types of information with differing biological coverage and would not always be expected to agree; therefore, prioritization thresholds were established independently for these two sets of screening values. An EAR_Chem_ value >10^−3^ was considered to have exceeded a prioritization threshold, consistent with previous similar analyses (Alvarez et al., 2021; Corsi et al., 2019) which found that using an EAR threshold of 10^−3^ resulted in a list of prioritized chemicals similar to that of traditional water‐quality benchmarks. A TQ value >0.1 was considered to have exceeded a prioritization threshold, similar to previous analyses (Corsi et al., 2019). The decision to use an EAR threshold of 10^−3^ and a TQ threshold of 0.1 was also based on hazard quotient comparisons, and it was part of the conservative approach used in this evaluation to provide an additional factor of safety due to 1) uncertainty in the derived benchmark values and 2) the assumption that the maximum stream concentrations were not observed in the present study (with only two to four samples collected per site). Further detail concerning the designation of EAR and TQ thresholds is provided in the Supporting Information. Despite the limitations of these hazard quotients, EAR and TQ values are well suited for comparisons across chemicals and across sites. Of the detected pharmaceuticals with EAR_Chem_ and/or TQ values above the respective thresholds, highest priority was assigned to pharmaceuticals observed above these thresholds at a minimum of 10% of sites monitored for their presence. Because analytes within Method 2 were not monitored at all sites, pharmaceuticals were prioritized according to the percentage of sites with threshold exceedances rather than the total number of sites with threshold exceedances to minimize potential biases.

The focus of the present study was pharmaceuticals; however, some of the analytes included in the laboratory analysis methods may not be considered strictly “pharmaceuticals.” Analytes which were closely related were considered “pharmaceuticals” for the purposes of the present study, including caffeine, nicotine, and artificial sweeteners. The occurrence of the related compounds, with respect to wastewater contributions, land cover, and hydrologic conditions, is comparable to that of other pharmaceuticals; and remediation actions to resolve environmental contamination resulting from their occurrence would likely be similar to those of other pharmaceuticals. However, among the 266 chemical analytes in the present study, nine chemicals were not considered to be closely related enough to be included as “pharmaceuticals,” including pesticides, corrosion inhibitors, plasticizers, and antioxidants (Supporting Information, Table SI‐2). These nine chemicals were excluded from most figures and further analysis in the present study; the term *chemical* has been specifically used when referring to aspects of the study that evaluated all of the monitored chemicals (both pharmaceuticals and nonpharmaceuticals). In addition, the nine nonpharmaceutical chemicals served as “contextual chemicals” and enabled comparisons between pharmaceuticals and other types of contaminants. Because these contextual chemicals were quantified from the same water samples as the pharmaceuticals and because alternative benchmarks were derived for contextual chemicals using the same methodology, the water chemistry data and alternative benchmarks for these chemicals serve as the closest points of comparison between pharmaceuticals and other types of contaminants in terms of detections, concentrations, and bioeffect potentials. Using this information puts the influence of all pharmaceutical analytes on the aquatic ecosystem into context relative to these nine other chemicals and emphasizes the fact that there are many other chemicals that may have as much influence as or more influence than pharmaceuticals.

### Site evaluation

Sites were evaluated by comparing the number of pharmaceuticals detected as well as the number of pharmaceuticals that exceeded various EAR and TQ thresholds from samples collected at each sampling location. Because chemicals within Method 1 were consistently monitored in all samples, this subset of data was used for direct comparison among sites. Additional information was compiled by site for chemicals in both Method 1 and Method 2 with the number of chemicals monitored included for context.

## RESULTS

### Occurrence and magnitude

In the present study, 113 samples were collected from 44 sites. From a total of 266 monitored chemicals, 110 pharmaceuticals and nine nonpharmaceuticals were detected (Supporting Information, Figure SI‐9 and Table SI‐2; Pronschinske et al., 2022). The number of chemicals (including nonpharmaceuticals) detected at a single site ranged from one to 81, with a minimum of three pharmaceuticals detected at 43 of the 44 sites in the present study. Forty or more chemicals were detected at 19 sites. There were 15 chemicals detected at 75% or more of the 44 sites, and 35 were detected at 50% of sites or more. Given that not all sites were monitored for Method 2 analytes as they were for Method 1, the chemicals that were detected at the most sites were metformin, caffeine, and atrazine, though many other chemicals occurred widely (Supporting Information, Table SI‐2).

The chemical classes with the highest average concentrations detected were artificial sweeteners, X‐ray contrast agents, and corrosion inhibitors. Individual chemicals within these classes had some of the highest mean detected chemical concentrations in the present study. For example, sucralose (artificial sweetener), iopamidol (X‐ray contrast agent), and 4‐methyl‐1H‐benzotriazole (corrosion inhibitor) were observed at mean concentrations >0.9 µg/L (Supporting Information, Figure SI‐9, Table SI‐2). Though many of these chemicals may not typically be considered pharmaceutical compounds, some of them were among the most abundant chemicals monitored in the present study.

**Figure 2:**
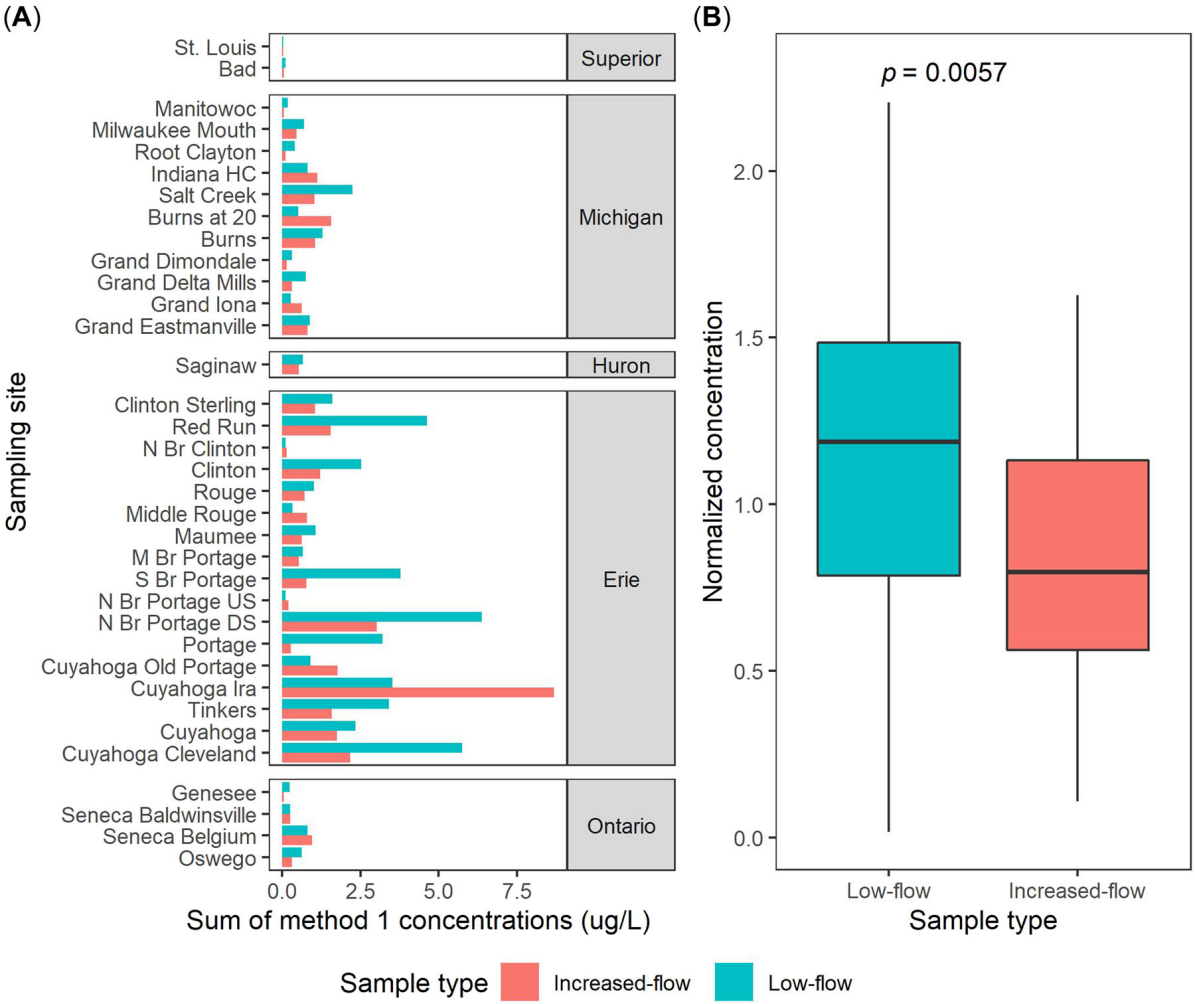
Summation of concentrations of Method 1 pharmaceuticals detected in Great Lakes tributary sampling sites, October 2017–September 2018. (**A**) Bar plot of concentrations grouped by site and separated based on the relative mean daily flow conditions present on sample collection dates. (**B**) Boxplots of summations of Method 1 pharmaceutical concentrations normalized by mean sample concentrations from individual sites. Mean normalized concentrations showed a significant difference (*p* < 0.05) between low‐flow and increased‐flow samples, according to Welch's two‐sample *t* test. Boxes depict the first through third quartiles; dark line, median; whiskers, data within 1.5 times the interquartile range. HC = Harbor Canal; N Br = North Branch; M Br = Middle Branch; S Br = South Branch; US = upstream; DS = downstream.

Pharmaceutical concentrations at most sites were lower during increased‐flow periods compared to low‐flow periods (Figure 2A). Sites in the upstream sections of the Cuyahoga River (downstream from Akron, OH, USA), the upstream section of the River Rouge, and the upstream section of Burns Waterway were among the exceptions: Concentrations at these three sites were considerably greater during periods of increased flow than during low‐flow periods, suggesting an event‐driven source of contamination in these watersheds. A broader view of this pattern is seen in the site‐normalized concentrations (Figure 2B). Median normalized sample concentrations during low‐flow periods were 49% greater than samples collected during increased‐flow periods (*p* < 0.05).

**Figure 3:**
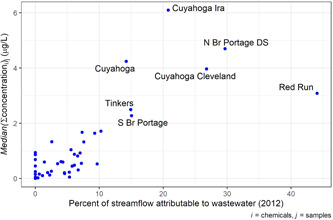
Relationship between relative contributions of wastewater‐treatment plants to streamflow and median sum of Method 1 pharmaceutical concentrations observed at each site from surface water monitoring results in Great Lakes tributaries, October 2017–September 2018. The correlation between these two variables is statistically significant (*p* < 0.05). Site abbreviations are included where *y* values are >2 µg/L. N Br = North Branch; S Br = South Branch; DS = downstream.

Effluent from WWTPs as a fraction of streamflow correlated positively with the Method 1 pharmaceutical concentrations observed in the samples (*p* < 0.05; Figure 3). On average, sites with higher fractions of WWTP effluent in their streamflow had higher sample concentrations; however, deviation from this relation suggested that consideration of other factors, such as certainty in WWTP effluent calculations, WWTP effluent composition (e.g., municipal vs. industrial), and additional sources of pharmaceuticals was also necessary. In eight of the watersheds, multiple sites were monitored to provide more detail on relative locations of pharmaceutical contamination sources and magnitudes (Supporting Information, Figure SI‐10). These individual watersheds reflect a general increasing pattern of pharmaceutical concentration in relation to the percentage of WWTP effluent but not in all cases.

### Pharmaceutical prioritization

**Figure 4:**
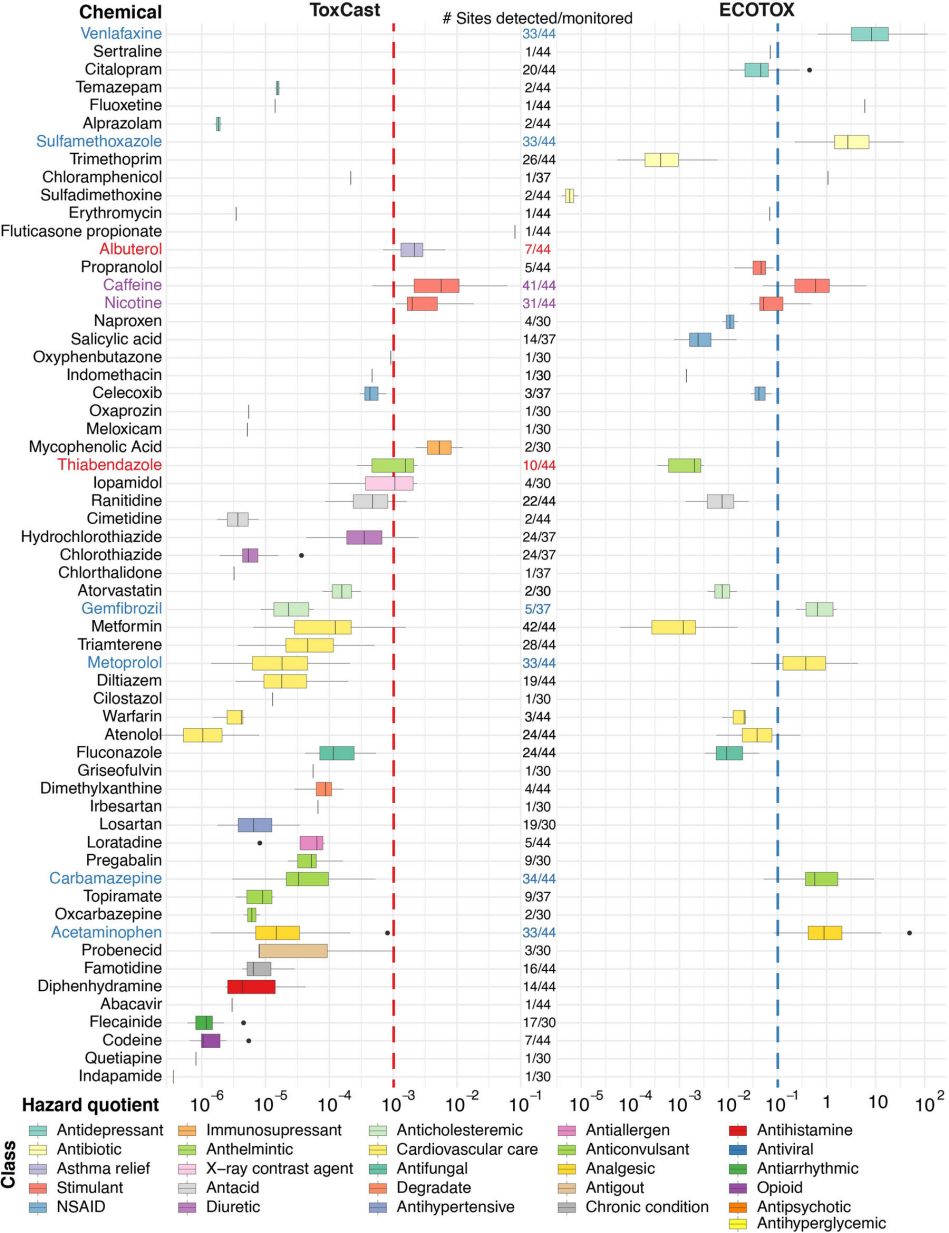
ToxCast exposure–activity ratios (EAR_Chem_; left) and ECOTOX‐derived toxicity quotients (TQs; right) for detected pharmaceuticals in samples collected from 44 Great Lakes tributaries, October 2017–September 2018. Both EAR_Chem_ and TQ values were computed using alternative water quality benchmarks and are types of hazard quotients. Pharmaceuticals are grouped by chemical class in descending order according to median EAR_Chem_. In cases where only one alternative water quality benchmark was available, the other was intentionally left blank. Pharmaceuticals not represented by either of the alternative water quality benchmark types were omitted. The EAR_Chem_ and TQ prioritization thresholds are represented by dashed lines at 10^–3^ and 10^−1^, respectively. High‐priority pharmaceutical labels are in red if prioritized according to ToxCast, blue if prioritized according to ECOTOX, and purple if prioritized based on both sets of benchmarks. Boxes depict the first through third quartiles; dark line, median; whiskers, data within 1.5 times the interquartile range; points, outliers. NSAID = nonsteroidal anti‐inflammatory drug.

Of the 110 pharmaceuticals detected in the present study, 50 had ToxCast data with relevant ACC values, and ECOTOX data were sufficient to develop benchmarks for 27 pharmaceuticals. By combining ToxCast and ECOTOX alternative benchmarks, 59 of the detected pharmaceuticals were represented by at least one alternative benchmark, allowing for the calculation of hazard quotients (EAR_Chem_ and TQ; Figure 4). The remaining 51 detected pharmaceuticals lacked sufficient information to compute hazard quotients (Supporting Information, Figure SI‐11 and Table SI‐13). The EAR and TQ values were also computed for the contextual chemicals; EAR values varied from 2 × 10^−8^ to 0.06, and TQ values varied from 7 × 10^−6^ to 262 for these chemicals (Supporting Information, Figure SI‐12).

Ten “high‐priority” pharmaceuticals that exceeded the EAR_Chem_ threshold of 10^−3^ or the TQ threshold of 0.1 at a minimum of 10% of monitored sites were identified (Table 2). One of the 10 high‐priority pharmaceuticals was represented in ToxCast but not in ECOTOX; two were represented in ECOTOX but not in ToxCast. Seven of the high‐priority pharmaceuticals had benchmark values from both data sources, but only two of these pharmaceuticals (caffeine and nicotine) exceeded thresholds from both sets of alternative benchmarks at 10% or more of the sites. In addition to the pharmaceuticals on the high‐priority list, others maintain the potential to elicit biological effects, including those crossing the hazard quotient thresholds (Figure 4), those with high detection frequencies, and/or those with concentrations which were not represented by alternative benchmarks (Supporting Information, Table SI‐13). The pharmaceuticals that exceeded the EAR threshold most frequently were caffeine and nicotine at 86% and 70% of sites, respectively; and these were the only two pharmaceuticals with EAR_Chem_ > 0.01 at more than one site (Figure 4). Six pharmaceuticals exceeded TQ thresholds at >65% of the sites (Table 2), and the greatest TQ values were attributable to venlafaxine, acetaminophen, and sulfamethoxazole, which were each detected at 75% of sites, exceeding the TQ threshold at a minimum of 70% of sites. The minimum ECOTOX benchmarks for venlafaxine and acetaminophen were both derived from the same study which reported increased embryo mortality resulting from exposures to these pharmaceuticals individually (Galus et al., 2013). The minimum ECOTOX benchmark for sulfamethoxazole was derived from a study that reported increased *p*‐aminobenzoic acid (pABA) in *Lemna gibba* due to sulfamethoxazole exposure. In addition, the study linked increased pABA to inhibition of folate biosynthesis, which, if disrupted, would lead to “severe metabolic and cellular dysfunction” (Brain et al., 2008).

**Table 2:** High‐priority pharmaceuticals, as determined by exceedance of the ToxCast exposure–activity ratio threshold of 10^−3^ or the ECOTOX toxicity quotient threshold of 0.1 at 10% or more of the sites from surface water monitoring results in Great Lakes tributaries, October 2017–September 2018

			Exceedances by site		
CAS	Chemical	Class	ToxCast EAR	ECOTOX TQ	Sites monitored
58‐08‐2	Caffeine	Stimulant	38	38	44
54‐11‐5	Nicotine	Stimulant	31	13	44
298‐46‐4	Carbamazepine	Anticonvulsant	0	33	44
723‐46‐6	Sulfamethoxazole	Antibiotic	–	33	44
93413‐69‐5	Venlafaxine	Antidepressant	–	33	44
103‐90‐2	Acetaminophen	Analgesic	0	31	44
51384‐51‐1	Metoprolol	Cardiovascular care	0	29	44
148‐79‐8	Thiabendazole	Anthelmintic	6	0	44
18559‐94‐9	Albuterol	Asthma relief	5	–	44
25812‐30‐0	Gemfibrozil	Anticholesteremic	0	5	37

CAS = Chemical Abstracts Service; TQ = toxicity quotient; EAR = exposure–activity ratio.

### Site evaluation

**Figure 5:**
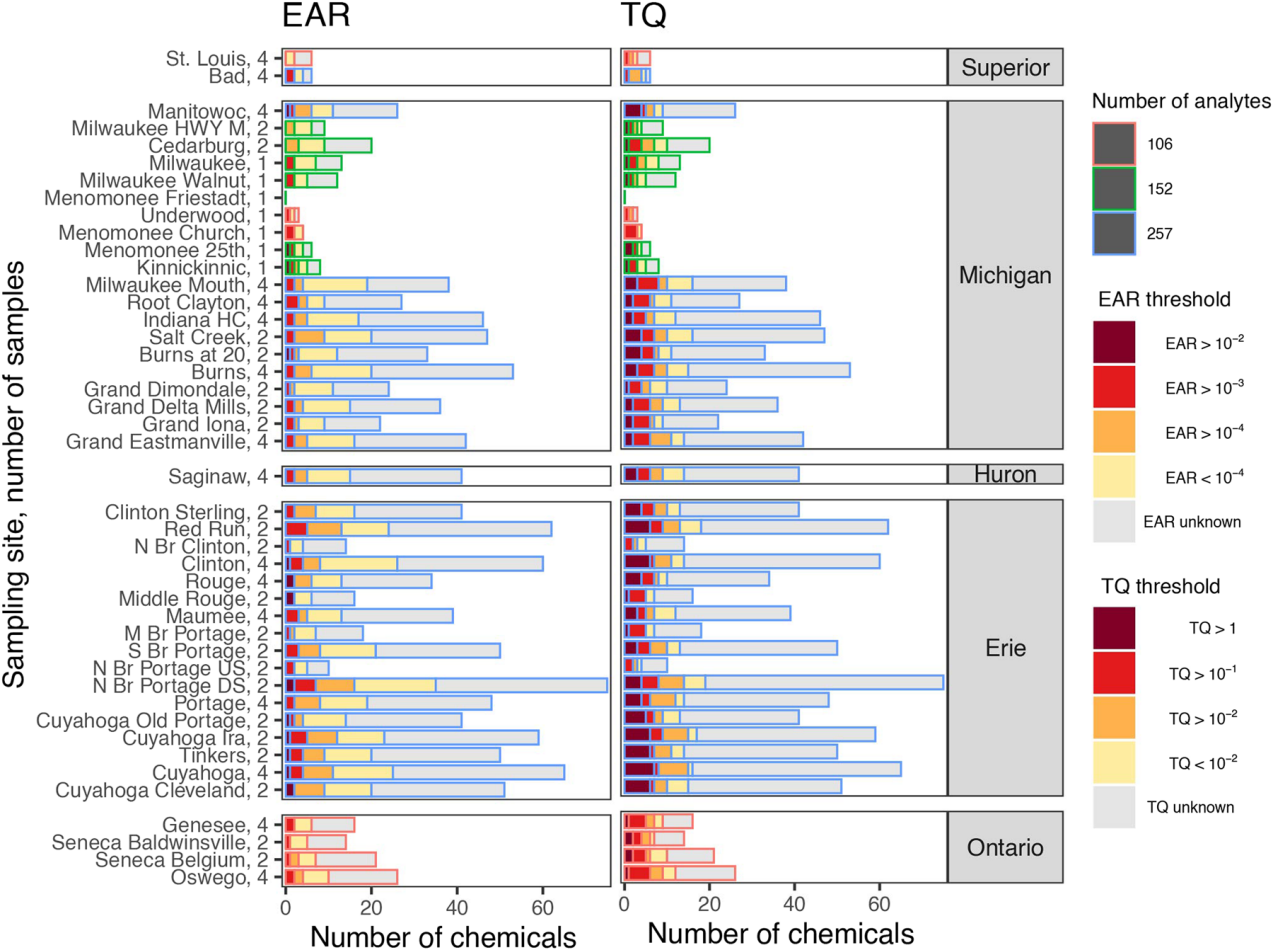
Pharmaceutical detections and exceedances of incremental hazard quotient thresholds for ToxCast exposure–activity ratios (left) and ECOTOX‐derived toxicity quotients (right), organized by sampling site. The number of pharmaceuticals detected at each site is represented by the combination of stacked bars (e.g., 60 pharmaceuticals were detected at Clinton). The number of pharmaceuticals monitored at each site is indicated by the border color of the respective bar (e.g., 257 pharmaceuticals were monitored at Clinton). The number of samples collected from each of the 44 Great Lakes tributary sampling sites, October 2017–September 2018, is listed following the site name. EAR = exposure–activity ratio; TQ = toxicity quotient; HWY = highway; HC = Harbor Canal; N Br = North Branch; M Br = Middle Branch; S Br = South Branch; US = upstream; DS = downstream.

Three watersheds (the Cuyahoga River, the Portage River, and the Clinton River), all within the Lake Erie basin, had sites with >60 detected pharmaceuticals; the greatest number detected (75) was at the North Branch Portage Downstream (N Br Portage DS) site downstream from Bowling Green, Ohio, USA (Figure 5). Only 10 pharmaceuticals were detected at the N Br Portage Upstream (US) site upstream from Bowling Green. Some of the sites with the lowest number of pharmaceuticals detected included those with little or no WWTP effluent, including five sites in the Milwaukee River watershed (within the Lake Michigan basin), the N Br Portage US site upstream from Bowling Green (within the Lake Erie basin), and the Bad and St. Louis Rivers (within the Lake Superior basin; Figure 5; Supporting Information, Table SI‐1).

**Figure 6:**
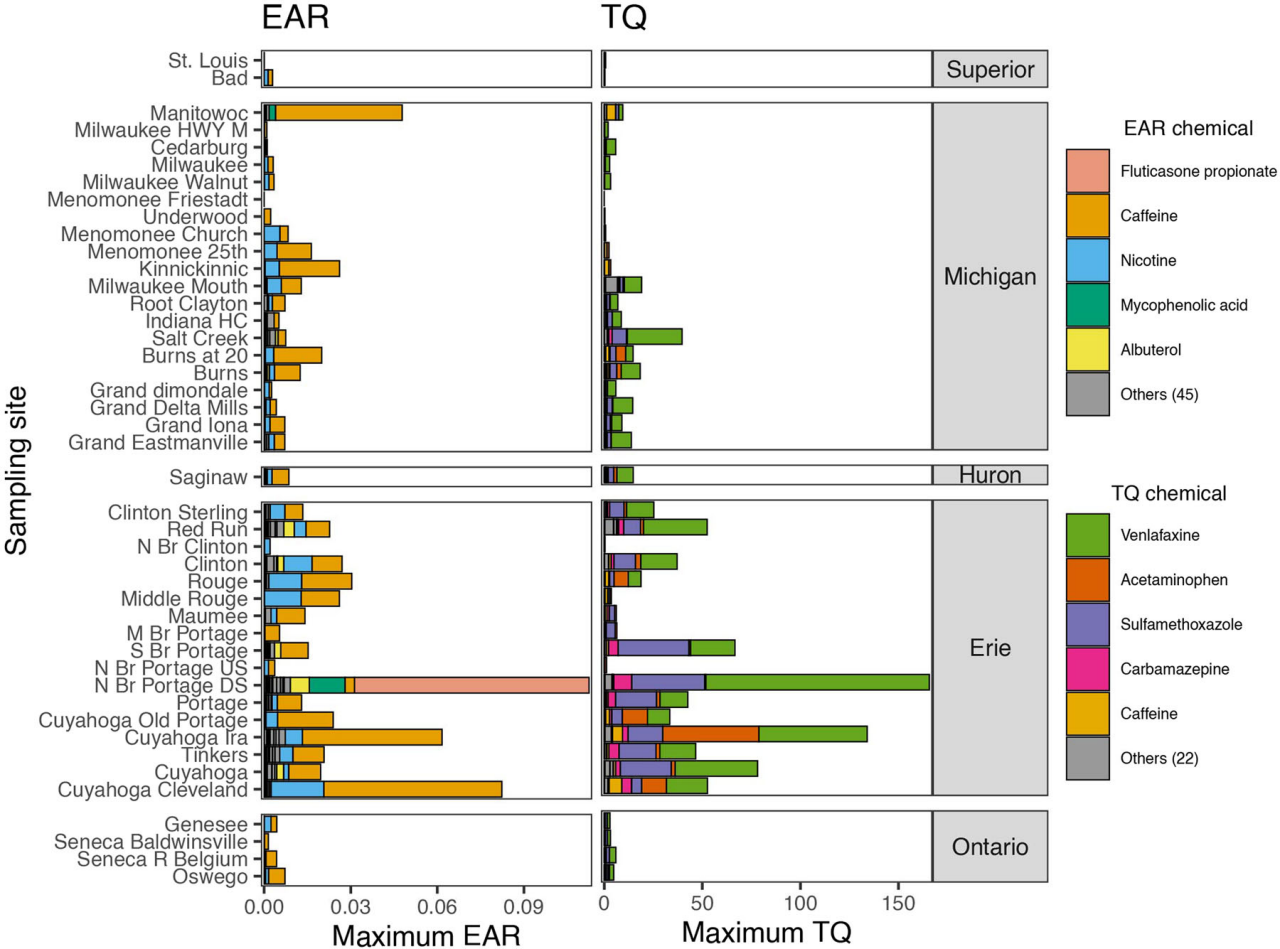
Pharmaceutical hazard quotients by site in the Great Lakes Basin from water samples collected in 44 Great Lakes tributary sampling sites, October 2017–September 2018. Sum of maximum ToxCast exposure–activity ratio values for all ToxCast assays (left) and maximum ECOTOX‐derived toxicity quotients for each pharmaceutical (right). The number of additional pharmaceuticals represented by each respective alternative benchmark is provided in parentheses next to “Others” in the key. EAR = exposure–activity ratio; TQ = toxicity quotient; HWY = highway; HC = Harbor Canal; N Br = North Branch; M Br = Middle Branch; S Br = South Branch; US = upstream; DS = downstream.

A mean of 44% of the detected pharmaceuticals by site were represented in ToxCast for computation of EAR values. Samples from all but one site had at least one pharmaceutical that exceeded the TQ and/or the EAR prioritization threshold (Figure 5): No pharmaceuticals were detected in samples from the Menomonee Friestadt site. Given that not all sites were monitored for all 257 pharmaceuticals, comparing only Method 1 pharmaceuticals may be beneficial in some cases (Supporting Information, Figures SI‐13 and SI‐14). Forty of the 44 sites included at least one pharmaceutical at a concentration that exceeded the EAR threshold. There were 14 locations which had samples with at least one pharmaceutical that exceeded the EAR threshold by an order of magnitude or more, all of which were collected in either the Lake Michigan or Lake Erie basin. In addition, samples from three sites (Red Run, Cuyahoga Ira, and N Br Portage DS—all located in the Lake Erie basin) had five or more unique pharmaceuticals that were detected at concentrations that exceeded the EAR prioritization threshold. Many of the EAR threshold exceedances were attributable to caffeine and nicotine (Figure 6). Exceedances of the EAR threshold for pharmaceuticals other than caffeine or nicotine occurred at 12 of the 44 sites.

The N Br Portage DS site had the greatest individual EAR_Chem_ value (fluticasone propionate), the greatest individual TQ value (venlafaxine), and the greatest sums of EAR_Chem_ and TQ values of the present study (Figure 6). Fluticasone propionate, a corticosteroid used for allergy and nasal symptoms, was only detected at N Br Portage DS. The Cuyahoga Cleveland, Cuyahoga Ira, and Manitowoc sites had the next three highest EAR_Chem_ values, all attributable to caffeine.

A mean of 41% of the detected pharmaceuticals by site were represented with ECOTOX‐derived benchmarks. Samples from 43 sites had at least one pharmaceutical that exceeded the TQ prioritization threshold. At least one pharmaceutical exceeded the TQ prioritization threshold by an order of magnitude or more in samples from 37 of the 44 sites, including sites in all Great Lakes basins except that of Lake Superior. Five or more pharmaceuticals were detected above the TQ prioritization threshold in samples from 29 of the 44 sites. The only watersheds without a site that had at least five pharmaceuticals with a TQ >0.1 were the Bad River and St. Louis River (in the Lake Superior basin).

Three individual pharmaceuticals were responsible for all values more than two orders of magnitude above the TQ threshold of 0.1: venlafaxine, acetaminophen, and sulfamethoxazole. These instances occurred in the Portage River, Cuyahoga River, Clinton River, and Grand River watersheds (in the Lake Erie basin) and in the Portage‐Burns Waterway (in the Lake Michigan basin).

## DISCUSSION

### Pharmaceutical prioritization

Means of prioritizing chemicals of concern are provided by EARs and TQs, but information availability varied by chemical. Prioritizing pharmaceuticals that had EAR_Chem_ >10^−3^ or TQ >0.1 at a minimum of 10% of sites monitored for the pharmaceutical resulted in a list of 10 pharmaceuticals. Of the remaining 247 pharmaceuticals, 147 were not detected, 49 were not bioactive and/or widespread, and 51 lacked sufficient data for alternative benchmark derivation. The prioritized pharmaceuticals fell into three different groups: 1) pharmaceuticals prioritized by either EAR or TQ threshold exceedances but lacking the other alternative benchmark (albuterol, sulfamethoxazole, and venlafaxine); 2) pharmaceuticals represented by both sets of alternative benchmarks but only prioritized by one (acetaminophen, carbamazepine, gemfibrozil, metoprolol, and thiabendazole); or 3) pharmaceuticals represented and prioritized by both sets of alternative benchmarks (caffeine and nicotine).

Pharmaceutical presence in groups one and two highlight the value of using ToxCast and ECOTOX evaluations together, which effectively reduces the number of chemicals with potential for biological effects that are overlooked when using only one method. Each of these toxicological data sources can serve to fill gaps that the other misses, but for some chemicals, sufficient information did not exist in either ToxCast or ECOTOX to adequately assess the potential effects. In fact, 51 of the 110 detected pharmaceuticals were not represented with alternative benchmarks from either ToxCast or ECOTOX. Of these, seven were present at 75% or more of the locations monitored for their presence (cotinine, lidocaine, fexofenadine, tramadol, bupropion, cetirizine, desvenlafaxine), and 12 additional pharmaceuticals were present at 50% or more of the locations monitored for their presence (methocarbamol; sucralose; sitagliptin; acesulfame; dextromethorphan; rac‐10,11‐dihydro‐10,11‐dihydroxy carbamazepine; desacetyl diltiazem; hydroxybuproprion; acyclovir; 10,11‐dihydro‐10‐hydroxy carbamazepine; valsartan; meprobamate; Supporting Information, Table SI‐13). Several of these high‐occurrence pharmaceuticals were also among those with the greatest concentrations in the present study (Supporting Information, Figure SI‐11 and Table SI‐2). This lack of toxicological information indicates a knowledge gap that could lead to mischaracterization of the potential for biological effects at any given location. For example, methocarbamol lacked sufficient information to derive alternative ToxCast or ECOTOX benchmarks; however, adverse effects, including altered hepatosomatic and aggression indices and increased hepatocyte vacuolization, were observed in fathead minnows in previous studies (Schoenfuss et al., 2016). These effects, observed at a concentration of 0.023 µg/L, could have resulted in methocarbamol being a top‐priority pharmaceutical if additional toxicological data had been available to derive an alternative benchmark for this pharmaceutical, suggesting that further toxicological assessment would be beneficial. Other pharmaceuticals of great interest for toxicological assessment may include widely occurring transformation products of priority pharmaceuticals, such as desvenlafaxine (product of venlafaxine) and cotinine (product of nicotine) which were detected at 75% and 86% of sites, respectively. Additional work to define the potencies of these pharmaceuticals could be valuable, based on their prevalence in Great Lakes tributaries and connection to high‐priority pharmaceuticals identified in the present study.

Caffeine is present in a wide variety of foods and beverages, and, as a component of tobacco, nicotine is consumed by direct and passive tobacco smoke inhalation (Buerge et al., 2003, 2008; Mackie et al., 2019; Mandel, 2002). Both pharmaceuticals are discharged into surface waters through treated wastewater effluent, sewage overflows, breaches in compromised sewage distribution systems, septic discharges, and possibly improper disposal of products containing these pharmaceuticals (Bradley et al., 2007; Buerge et al., 2003, 2008; Olds et al., 2018; Roder Green et al., 2014; Rutsch et al., 2007). Treatment of caffeine and nicotine in wastewater systems is relatively effective at 65%–99% removal (Ekpeghere et al., 2018; Kosma et al., 2014), but given the level at which they are present in sewage, what remains is still enough to result in their frequent detection in surface water systems and their use as indicators of anthropogenic activities (Bradley et al., 2017; Buerge et al., 2003, 2008; Chen et al., 2002; Kosma et al., 2014). In addition, cigarette butts are a common item of litter, with one study reporting a litter rate of 77% of all cigarettes smoked in public areas within cities (Patel et al., 2013). Nicotine is leached from the cigarette butts, serving as a source to nearby receiving waters (Roder Green et al., 2014).

The present study indicated widespread occurrence and exceedance of EAR and TQ thresholds by caffeine and nicotine, and both have been evaluated previously for their potential effects on aquatic organisms (Li et al., 2020; Oropesa et al., 2017). In a review of biological effects of caffeine, the predicted NOEC was 0.5 µg/L in a regeneration study with the polychaete *Diopatra neapolitana* (Li et al., 2020; Pires et al., 2016). Toxicity testing of *Vibrio fischeri*, *Pseudokirchneriella subcapitata*, *Thamnocephalus platyurus*, and *Daphnia magna* indicated that nicotine poses a potential risk to aquatic ecosystems, with measured effects on *Daphnia magna* reproduction at concentrations as low as 10 µg/L and a NOEC of 1 µg/L (Oropesa et al., 2017). The aforementioned study did not consider the presence of transformation products associated with nicotine such as cotinine. These NOECs are only slightly above the highest concentrations observed for caffeine and nicotine in the present study. Given the uncertainties in NOEC derivation and that maximum concentrations for each stream were not likely to be captured in the periodic sampling for the present study, there does appear to be the potential for effects from these two pharmaceuticals.

The remaining eight pharmaceuticals designated in the present study as a “priority” represent a diverse set of pharmaceutical use classes intended to address a variety of human health concerns (Table 2). These pharmaceuticals are all prescription drugs except for the nonsteroidal anti‐inflammatory drug acetaminophen, which is available over the counter for pain and fever reduction as well as being compounded with other pharmaceuticals in prescription and over‐the‐counter medications. As described above for caffeine and nicotine, these pharmaceuticals are primarily introduced to receiving water in various forms of treated or untreated waste. Wastewater‐treatment effectiveness can vary widely for these pharmaceuticals, with removal efficiencies varying from no removal to complete removal depending on the pharmaceutical and the treatment system (Greenham et al., 2019; Matongo et al., 2015; Metcalfe et al., 2010; Sánchez Peréz et al., 2014; Zhang et al., 2008; Zhou et al., 2009). In addition, several of these pharmaceuticals are administered to pets and livestock (Plumb, 2018), and thiabendazole is also used as a fungicide in agricultural applications (Lombardi et al., 2003). Runoff of pet waste, barnyards, and land application of manure are well‐recognized sources of contamination to surface water (Ahmed et al., 2020; Clarke & Cummins, 2015; Ervin et al., 2014; Feng et al., 2018; Khaleel et al., 1980; Müller et al., 2020). All eight of these pharmaceuticals have been detected in surface waters from multiple regions of the world at concentrations similar to or exceeding the concentrations observed in the present study (Bradley et al., 2017; Choi et al., 2008; Fick et al., 2009; Furlong et al., 2017; González Alonso et al., 2010; Hughes et al., 2013; Johnson et al., 2015; Jux et al., 2002; Matongo et al., 2015; Ramaswamy et al., 2011; Schultz & Furlong, 2008; Sun et al., 2015; Zhou et al., 2009).

Exceedance of EAR and TQ thresholds for these eight pharmaceuticals occurred over multiple sites, with exceedances in tributaries for each of the five Great Lakes and over a large variation of watershed characteristics (Figure 5 and Table 2; Supporting Information, Table SI‐1). The water‐quality benchmarks used for these eight pharmaceuticals originated from a diverse set of assays as well. Test organisms for endpoints used for ECOTOX benchmark derivation included freshwater mussels, amphibians, water fleas, duckweed, and fish and included apical (venlafaxine, metoprolol, thiabendazole, acetaminophen) as well as genetic (carbamazepine), biochemical (sulfamethoxazole), and cell‐level (gemfibrozil) assays. The minimum ACC values from ToxCast used to compute EAR values were from in vitro assays targeted at measurement of messenger RNA induction (thiabendazole, ToxCast assay CLD_CYP1A1_24hr) and signal transduction pathways for cellular function, gene transcription, and protein expression (albuterol, ToxCast assay TOX21_TSHR_wt_ratio). A direct relation to ecological relevance for the suite of ECOTOX and ToxCast assays leading to these TQ and EAR assessments is clearer for some (apical endpoints) than for others (in vitro assays).

#### Comparison to contextual chemicals

All nine nonpharmaceutical contextual chemicals were detected in the present study (Supporting Information, Table SI‐2). Often, the EAR and TQ values calculated for pharmaceuticals were substantially less than values from three of the contextual chemicals: bisphenol A (BPA), fipronil, and atrazine. Hazard quotients for these three chemicals were among the greatest values observed in the present study (Supporting Information, Figure SI‐12). Bisphenol A is used in numerous commercial applications and is known to be a disruptor of endocrine systems in humans as well as aquatic species at environmentally relevant concentrations (Flint et al., 2012). Fipronil is an insecticide that impacts the nervous system of insects and is used to kill fleas, ticks, ants, beetles, and other insects, with a range of uses from agricultural to household pet care products. Fipronil is toxic to fish and aquatic invertebrates and has been reported to elicit adverse effects at concentrations below USEPA chronic benchmarks for invertebrates (Gibbons et al., 2015; Miller et al., 2020). Atrazine is an herbicide that has a wide range of potential adverse effects to aquatic life, including reproductive dysfunction in fish and reduced growth in algal species (Richter et al., 2016; Stratton, 1984), and it has previously been identified as a chemical of concern in Great Lakes tributaries (Ankley et al., 2021; Corsi et al., 2019). As an herbicide, atrazine has the potential to impact aquatic and wetland vegetation and inhibit water‐quality improvements from wetland ecosystems (Graymore et al., 2001). Atrazine has been restricted or banned in several countries (Bethsass & Colangelo, 2006; Graymore et al., 2001; Wolf & Nowak, 1996). The mean hazard quotients of most pharmaceuticals in the present study were lower than those of BPA, fipronil, and atrazine; however, many pharmaceuticals had hazard quotients similar to the remainder of the contextual chemicals—piperonyl butoxide, mecoprop, and triazoles (corrosion inhibitors). Although pharmaceuticals maintain the potential to adversely affect aquatic organisms, other chemicals are ostensibly more harmful. In particular, BPA and atrazine serve as direct evidence of additional chemicals and classes with bioeffect potentials that exceeded the greatest hazard quotients attributable to pharmaceuticals monitored in the present study, thus emphasizing the importance of considering other contaminant classes.

### Site evaluation

Combining the results from pharmaceutical detections and analysis of EAR and TQ computations indicated that pharmaceuticals are present in most watersheds, but the potential for biological effects was generally greatest in the western Lake Erie watersheds, southern Lake Michigan watersheds, and the one site monitored in Lake Huron (Saginaw). Given that the N Br Portage DS site had the most pharmaceuticals detected, the greatest EAR values, and the greatest TQ values, this site had the greatest potential for biological effects of all sites in the present study (Figures 5 and 6). This site was downstream from Bowling Green, Ohio, USA. The site upstream from Bowling Green had much lower detection rates and much lower potential for biological effects based on EAR and TQ analyses. Urban runoff and the WWTP effluent from the city of Bowling Green enter the stream between the two sites. The treated wastewater effluent as a percentage of streamflow increases from 0.0% to 30% from upstream to downstream (Table 1). The site with the least influence from pharmaceuticals was in the Milwaukee River watershed (Menomonee Friestadt), which drains an area primarily consisting of agricultural and wetland land cover with no treated WWTP effluent and the lowest human population density in the present study (Table 1).

Most other watersheds had exceedances of EAR and TQ thresholds to varying degrees. Some of this variability is explained by WWTP effluent presence: the watersheds with the lowest pharmaceutical concentrations were those with a low degree of WWTP effluent, and sites with the greatest concentrations had high degrees of WWTP effluent, resulting in significant, positive correlation between WWTP contributions and pharmaceutical concentrations (Figure 3; Supporting Information, Table SI‐1). However, the Red Run site (which had the highest WWTP‐streamflow fraction) had only moderate pharmaceutical concentrations, which may be due to the composition of the wastewater at the site (Figure 3). Red Run had the highest percentage of urban land cover among all sites in the present study (Supporting Information, Table SI‐1); however, much of that land cover is industrial. Red Run had the second highest percentage of *Urban: High intensity* land cover in its watershed: This land cover class may include commercial and/or industrial areas. If large volumes of wastewater are produced by nonpharmaceutical industries, such as the automotive manufacturing facilities surrounding Red Run, those wastewaters may actually serve to dilute pharmaceutical concentrations in streams. As a result, relatively low pharmaceutical concentrations may be expected in samples collected from sites with highly industrial WWTP effluent. In addition, the streamflow at Red Run was elevated at the times that both samples were collected; this increased flow and runoff likely diluted pharmaceutical concentrations further.

Although many of the watersheds showed a general positive relationship between wastewater contributions and pharmaceutical concentrations, several of the watersheds (Cuyahoga, Milwaukee, and Rouge) illustrate that this relation is more complex than a simple correlation but likely reflects the influence of additional sources (Supporting Information, Figure SI‐10). This variability could be the result of numerous factors, beginning with uncertainty introduced by a lack of WWTP effluent data on the same date as sampling and including each aspect of the full life cycle of wastewater (collection, treatment, disposal, and reuse) and from other urban and agricultural nonpoint runoff sources. Upstream from the wastewater‐treatment process, conveyance systems that transport raw wastewater to WWTPs are often found to be imperfect, with failing infrastructure causing leaks and misconnections periodically during the construction process (Rutsch et al., 2006). As discussed for several of the highlighted pharmaceuticals in the present study, treatment effectiveness can vary depending on the type of treatment, having an immediate impact on the occurrence and concentration of pharmaceuticals present in streams that receive WWTP effluent (Nguyen et al., 2021; Pal, 2018). Treated wastewater sludge that is land‐applied for agricultural purposes has been shown to have potential to contaminate nearby streams (Ghirardini & Verlicchi, 2019; Verlicchi & Zambello, 2015). Discharge from improperly functioning septic systems can also contribute contamination by pharmaceuticals to receiving waters (Ferguson et al., 2003; Peed et al., 2011). In addition to these human wastewater sources, pharmaceuticals that are used in veterinary applications can be present in agricultural runoff that contains livestock manure (Motoyama et al., 2011; Sim et al., 2013) or in urban and rural stormwater that contains pet waste (Müller et al., 2020). In the present study, several sites had <1% WWTP effluent as a percentage of mean streamflow but still had pharmaceuticals present, including the St. Louis and Bad River sites (in the Lake Superior basin), five sites within the Milwaukee River watershed and the Indiana Harbor Canal site (in the Lake Michigan basin), the N Br Portage US and both River Rouge sites (in the Lake Erie basin), and Genesee (in the Lake Ontario basin). Land cover in these watersheds varies from highly urban to highly agricultural to relatively undeveloped. This verifies the potential for contribution of pharmaceutical contamination from a diversity of sources other than WWTP effluent.

## CONCLUSION

Monitoring data from the present study and corresponding toxicological information indicated that pharmaceuticals were present in nearly all samples monitored and that there was potential for biological effects of pharmaceuticals on aquatic organisms. Given that no formally established water‐quality benchmarks were available for the detected pharmaceuticals, monitoring results were compared with alternative benchmarks—gathered from the ToxCast database and derived from endpoints collated from the ECOTOX Knowledgebase—to gain a biological perspective on exposure to detected chemicals. These methods were effective at identifying 10 pharmaceuticals as a high priority, but still, the availability of toxicological data for evaluating these results was limited and remains one of the limiting factors in prioritizing pharmaceuticals and other environmental contaminants. The ToxCast‐ and ECOTOX‐derived benchmarks represented just over half of the detected pharmaceuticals. This left 51 detected pharmaceuticals without a basis for the evaluation of potential biological effects, many of which were detected at more than half of the sites. Additional toxicological work is necessary to generate sufficient information to thoroughly evaluate potential biological effects from pharmaceuticals. Pharmaceutical prevalence was shown to be greater during low‐flow periods than during periods of increased flow. Presence of WWTP effluent was significantly correlated to pharmaceutical concentrations, but the occurrence of pharmaceuticals at sites where little to no WWTP effluent was present with a diversity of land‐cover profiles indicated that additional sources also contributed pharmaceutical contaminants to tributaries of the Great Lakes. The results of the present study may help inform resource managers of the relative toxic potentials of the evaluated pharmaceuticals and elucidate when and where pharmaceutical concentrations may be highest.

## Supporting Information

The Supporting information are available on the Wiley Online Library at https://doi.org/10.1002/etc.5403.

## Disclaimer

Any use of trade, product, or firm names is for descriptive purposes only and does not imply endorsement by the US government.

## Author Contributions Statement

**Steven R. Corsi**: Conceptualization; Funding acquisition; Project administration; Supervision. **Matthew A. Pronschinske**, Data curation; Formal analysis; Methodology; Validation; Visualization; Writing—original draft; Writing—review & editing. **Michelle A. Nott**: Data curation; Formal analysis; Visualization; Writing—review & editing. **Steven R. Corsi**: Formal analysis; Methodology; Visualization; Writing—original draft; Writing—review & editing. **Laura A. DeCicco**: Formal analysis; Methodology; Software; Visualization. **Peter L. Lenaker**: Investigation. **Edward T. Furlong**: Investigation; Methodology; Resources; Validation; Writing—original draft. **Gerald T. Ankley, Brett R. Blackwell, Daniel L. Villeneuve**: Writing—review & editing.

## Supporting information

This article includes online‐only Supporting Information.

Supporting information.

Supporting information.

## Data Availability

Data and metadata are provided in the Supporting Information of the manuscript, and can be found online at https://doi.org/10.5066/P9YIT6O9, and https://waterdata.usgs.gov/nwis.
