# Supplementary material for: Prioritizing Pharmaceutical Contaminants in Great Lakes Tributaries Using Risk‐Based Screening Techniques
Source: Environ Toxicol Chem. 2022 Jul 21;41(9):2221–39. doi: 10.1002/etc.5403 (PMC9542422; doi:10.1002/etc.5403)
Supplement: Supplementary file 1 — Supporting information. [file ETC-41-2221-s001.DOCX]

Prioritizing Pharmaceutical Contaminants in Great Lakes Tributaries Using Risk-Based Screening Techniques: Supplemental Information

Table of Contents

[Disclaimer 2](#_Toc103252700)

[GIS methods 2](#_Toc103252701)

[Sample collection 2](#_Toc103252702)

[Laboratory analysis 3](#_Toc103252703)

[Blank and duplicate samples 5](#_Toc103252704)

[Screening values 5](#_Toc103252705)

[ToxCast EAR derivation 5](#_Toc103252706)

[Screening for potential pathway-based effects 6](#_Toc103252707)

[Benchmark derivation 6](#_Toc103252708)

[Figures 8](#_Toc103252709)

[Figure SI-1 8](#_Toc103252710)

[Figure SI-2 9](#_Toc103252711)

[Figure SI-4 11](#_Toc103252712)

[Figure SI-5 12](#_Toc103252713)

[Figure SI-6 13](#_Toc103252714)

[Figure SI-7 14](#_Toc103252715)

[Figure SI-8. 15](#_Toc103252716)

[Figure SI-9 16](#_Toc103252717)

[Figure SI-10 17](#_Toc103252718)

[Figure SI-11 18](#_Toc103252719)

[Figure SI-12 19](#_Toc103252720)

[Figure SI-13 20](#_Toc103252721)

[Figure SI-14 21](#_Toc103252722)

[References: 22](#_Toc103252723)

# Disclaimer

Any use of trade, product, or firm names is for descriptive purposes only and does not imply endorsement by the U.S. Government.

# GIS methods

A geographic information system (GIS) was used to determine watershed-scale characteristics for all sites (Table 1 and Table SI-1). Methods are described briefly here; additional detail can be found in Baldwin et al. (2016), which used similar methods. Watershed boundary linework was derived from the Watershed Boundary Dataset (WBD) (U.S. Department of Agriculture-Natural Resources Conservation Service et al. 2009). Land cover percentages were determined using the 2016 National Land Cover Database (NLCD) (Yang et al. 2018; Jin et al. 2019; Wickham et al. 2021), and aggregated to several Level I categories. Population statistics were determined from 2010 state-based block datasets, and associated calculations were based on the assumption that measured populations were evenly distributed within each area (U.S. Census Bureau Geography Division 2010). Wastewater treatment plant (WWTP) contributions were determined from two derivative datasets of the U.S. Environmental Protection Agency National Pollutant Discharge Elimination System (NPDES): one from the International Joint Commission (Laitta 2016), and one from the USGS Spatially Referenced Regression on Watershed Attributes (SPARROW) modeling group (Maupin and Ivahnenko 2011). Both datasets were reflective of 2012 conditions, and in both cases, data had been updated and corrected following download; where the same facility (i.e., same NPDES) existed in both datasets, preference was given to the IJC dataset. WWTP data were used in conjunction with 2012 streamflow data from NWIS to assess the percentage of streamflow attributable to WWTP effluent. Sampling sites without streamflow data were estimated using drainage-area-weighted values from the nearest available stream gages. Distance-weighting of WWTP contributions was done using the catchment shapefiles and value-added attribute tables provided with the medium-resolution NHDPlus v. 2 dataset (U.S. Environmental Protection Agency and U.S. Geological Survey 2012). Manual adjustments were necessary in a few cases and were based on manual measurements made in consultation with aerial imagery.

# Sample collection

Equal-width increment (EWI) sampling was employed at each sampling site to collect a composite water sample from a lateral transect of the stream (U.S. Geological Survey 2006: 4). To prevent the contamination of samples from sampling equipment, Teflon equipment (sample bottle, sample nozzle, and churn) was used. Equipment was cleaned with phosphate-free soap (Liquinox®) and tap water, sprayed with methanol, rinsed twice with deionized water, and finally rinsed with pesticide-grade blank water (MilliporeSigma OmniSolv®, WX0004-1) according to methods described in the USGS National Field Manual (U.S. Geological Survey 2004: 3). Prior to collection, all sampling equipment was field rinsed three times with water from the sampling site. Subsamples were collected from points along the lateral stream transect and composited into the Teflon churn according to methods described in the USGS National Field Manual (U.S. Geological Survey 2006: 4). Next, the sample was processed by thoroughly churning the composited sample and drawing a final subsample from the Teflon churn into a 20 mL plastic syringe. A 0.7 µm glass fiber filter (Whatman® GMF, 6825-2517) was attached to the syringe and pre-conditioned by discharging 10 mL of sampled water before discharging the remaining 10 mL sample into a 20 mL, baked, amber-glass vial according to methods described in the USGS National Field Manual (U.S. Geological Survey 2002: 5). The samples were stored in the absence of light between 0 and 4 degrees Celsius and arrived at the laboratory for analysis within 48 hours of their collection.

Duplicate and blank samples were collected to ensure the quality of water concentration data. For duplicate samples, after the regular sample was drawn from the churn, a second sample was drawn from the same churn. Duplicate samples were processed and analyzed using methods identical to regular samples. Blank samples were collected by passing pesticide-grade blank water (instead of sampled stream water) through clean sampling equipment (sample bottle, sample nozzle, and churn). The blank sample was processed and analyzed using methods identical to regular samples. The methods for collecting duplicate and blank samples are further described in the USGS National Field Manual (U.S. Geological Survey 2006: 4).

# Laboratory analysis

The methods used in this study to identify and quantify pharmaceutical compounds is described more fully in Furlong et al. (2014). A brief summary of the two methods follows.

Filtered aliquots of water samples collected during this study were anlayzed for pharmaceuticals and related compounds using two methods. The first method (Furlong et al. 2014), includes 109 analytes, most of which are pharmaceutical compounds (Table SI-2) and was used for all samples collected. In this method, a 100-microliter aliquot of the filtered water sample is directly injected into a high-performance liquid chromatograph (HPLC) coupled to a triple quadrupole mass spectrometer (MS/MS) using electrospray ionization operated in the positive ion mode. The analytes are separated using C-18 reversed-phase HPLC column with a gradient of formic acid/ammonium formate-modified water and methanol. Multiple reaction-monitoring; MRM was used to confirm the identity of each analyte and to quantify each analyte, relative to one of a suite of isotopically labeled pharmaceutical analogues added prior to analysis. Method reporting limits (RLs; determined in reagent water) for individual chemicals ranged between 2 and 270 nanograms per liter (ng/L) during the period these samples were analyzed; the USGS National Water Quality Laboratory annually assesses RLs using the procedure of ASTM International (2016), as documented in Williams et al. (2015). The median RL for all analytes was 30 ng/L. The majority of method detection limits (MDLs) for this method, as defined by the 25^th^ and 75^th^ percentiles of MDL distribution, fell between 13 and 80 ng/L.

The second method to determine pharmaceutical compounds in this study complements and expands the number of chemicals beyond the method of Furlong et al. (2014). This method is functionally similar to the first, that is, it uses direct aqueous injection of a filtered water sample, separates the chemicals using HPLC directly coupled to MS/MS, and uses MRM to produce two unique precursor-product ion pairs for identification and quantitation of individual chemicals. However, there are several important differences in this newer method:

1. Two separate instrumental analyses are used for the new method, the first using negative electrospray ionization (ESI) and the second using positive ESI. The negative ESI analysis was developed to expand the classes of pharmaceutical compounds determined to include statins, nonsteroidal anti-inflammatory drugs (NSAIDs) and other compounds that preferentially ionize more efficiently, and are thus more sensitively detected and identified, under negative ESI conditions. This method can be used to determine 158 chemicals (all but one of which had not been previously determined under Method 1): 49 under negative ESI conditions and 109 under positive ESI conditions. One chemical (lorazepam) is shared between the Method 1 and Method 2 positive ESI.
2. A different reverse phase HPLC column is used for both instrumental analyses to achieve separation of the chemicals prior to ionization. The column used in this method is an Agilent Technologies Poroshell HPH-C18, 2.7 μm particle size, 4.6 mm inside diameter by 100mm length (catalog number G1958-67098).
3. Different HPLC mobile phases and gradients are used. The aqueous mobile phase (Mobile Phase A) is organic-free water modified with aqueous 1 molar ammonium fluoride and 1 molar ammonium hydroxide to final mobile phase concentrations of 0.2 millimolar for each, and the organic mobile phase (Mobile Phase B) is 90:10 Methanol:2-propanol modified with aqueous 1 molar ammonium fluoride and 1 molar ammonium hydroxide) to final mobile phase concentrations of 0.2 millimolar for each. The mobile phase gradient for both the negative and positive ESI analyses is shown in Table SI-14.

The concentration of the qualitatively identified pharmaceutical was determined using isotope dilution standards specific to the positive and negative ionization conditions for the two instrumental analyses used in method 2 and applied as in Furlong et al. (2014). Interim reporting limits (IRLs) were initially determined using the method detection limits calculation procedure of the U.S. Environmental Protection Agency (2005). This determination was based on a single set of determinations made at the start of the use of this method. A more robust determination of method reporting limits (RLs) was made using multiple data sets collected across the operational range of the method and across a six-month period coincident with the analysis of the samples collected for this study. These method RLs were determined by the method of ASTM International (2016). These RLs were then compared to method detection limits calculated from the same data set using the procedure of the U.S. Environmental Protection Agency (2005), and the more conservative RL was applied . This combined reporting limit process is described more fully in Williams et al. (2015). The Pharmaceutical-specific IRLs and method RLs are listed in Table SI-3. IRLs are reported because they were used for data evaluation of the study. The method RLs are reported because they are more reflective of overall long-term method performance and provide an evaluation baseline across the course of this study. The median IRL for all analytes was 92 ng/L. The majority of IRLs for this method, as defined by the 25th and 75th percentiles of IRL distribution, fell between 24 and 160 ng/L. In comparison, the median method RL for all analytes was 20 ng/L, and the majority of method RLs for this method, as defined by the 25th and 75th percentiles of method RL distribution, fell between 20 and 81 ng/L.

Note that any detected pharmaceutical that met qualitative identification criteria used for this method (European Commission 2002) but whose concentration was below the IRL was reported as an estimated concentration, reflecting somewhat greater uncertainty around the reported concentration than for concentrations above the IRL. Note that some chemicals were routinely reported as estimated values regardless of concentration because of variation in method performance. Criteria for qualifying concentrations as estimates are provided in Childress et al. (1999).

# Blank and duplicate samples

There were 11 blank samples collected and analyzed for Method 1 analytes, and 10 blanks samples for Method 2. All blank sample detections are included in Table SI-4. Nicotine was detected in three of the blank samples, but it was not excluded from sample results because this screening-level study sought to identify potentially concerning chemicals despite uncertainties. There were 20 duplicate samples collected and analyzed for Method 1 analytes, and 81 duplicate samples for Method 2; duplicate samples were collected at the same time as regular samples for comparison purposes. More duplicate samples were collected for the verification of Method 2 analytes because the laboratory analysis methods for these chemicals were in development at the time of sample collection. From the 10,991 comparisons (Table SI-5), 10,275 comparisons had no detections in either sample of the regular/duplicate pair. There were 560 comparisons in which the same chemical was detected in both samples of the pair; among these comparisons, the median relative percent difference (RPD) was 18%. There were 156 comparisons where a chemical was detected in one sample but not its pair; as in Baldwin et al. (2016), RPD was computed using the detected chemical concentration and one-half of the reporting level, and the median RPD was 86%.

# Screening values

## ToxCast EAR derivation

ToxEval (DeCicco et al. 2018) was used to gather assay results from version 3.2 of the ToxCast Databased (U.S. Environmental Protection Agency 2017) and evaluate the potential biological activities associated with each chemical detected in water samples collected for this study. Data analysis routines to define exposure-activity ratio (EAR) values for detected chemicals were similar to previously published methods (Blackwell et al. 2017; Corsi et al. 2019). ToxEval was used to filter ToxCast data by removing flagged data and cleaning endpoint information. Additionally, dose-response curves were examined; assays which seemed unreliable or responses which seemed to be of questionable quality were removed. Excluded assays or chemical-assay pairs are listed in Table SI-8.

## Screening for potential pathway-based effects

Extrapolating EAR information for application to environmental concentrations can be complex. For example, current information does not include correction for chemical partitioning in the assay system (e.g., free versus bound chemical in a test well), so actual biological activity in vitro or in situ may differ from this. However, it does provide a value that effectively normalizes for relative concentration detected in the environment and relative potency to elicit a specific biological effect. Thus, the EAR value is suitable for relative ranking and prioritization. Still, it is recognized that not all ToxCast assays used in this analysis are likely to be relevant to ecological species even though many of them target biological activities that are conserved among species. A complete evaluation of ToxCast assays for ecological relevance is currently not available, so the EAR approach used here is conservative in this respect.

## Benchmark derivation

The process for benchmark development in this study was based on the process used by Hull et al. (2015), with modifications. Modifications included assigning a higher application factor (AF) value to Acute Effect endpoints, not considering duration as a factor in order to retain more ECOTOX studies, and including acute endpoints in the test species diversity AF assessment. Using this framework, a set of three benchmarks (No Effect, Low Effect, Acute Effect) were developed for each chemical depending on ecotoxicity data availability within ECOTOX. Unlike Hull et al. (2015), study duration was not considered as a factor when defining benchmark types. In the benchmark development process, minimum concentration values were divided by application factors depending on characteristics of the chemical tested and the study performed. The following data was required for the development of a comprehensive application factor: endpoint code, chemical persistence, and the variety of species tested in the toxicity studies gathered from the ECOTOX database.

$$Benchmark= \frac{Minimum endpoint subset concentration\left( \mu g/L \right)}{\left( \mathrm{AF}_{\mathrm{Endpoint}}\times\mathrm{AF}_{\mathrm{Species}}\times\mathrm{AF}_{\mathrm{Persistence}} \right)}$$

The ECOTOX results were assigned to one of the three benchmark types (No Effect, Low Effect, and Acute Effect) depending upon the ECOTOX-reported endpoint code. The minimum concentration from a NOEC/NOEL or EC/LC ≤ 10 endpoint was generally used to define the No Effect benchmark type for each chemical. Similarly, the minimum concentration from a LOEC/LOEL or EC/LC 10 < x < 50 endpoint was generally used to define the Low Effect benchmark type for each chemical. Finally, the minimum concentration from an EC/LC50 endpoint was generally used to define the Acute Effect benchmark type for each chemical. No Effect and Low Effect benchmark types earned an ${AF}_{endpoint}$ of 10 (Hull et al. 2015). Unlike Hull et al. (2015), Acute Effect benchmark types earned an elevated ${AF}_{endpoint}$ of 100 due to the assumption that lower-level effects may be elicited at concentrations considerably lower than those which provoke acute effects.

Subsequently, an application factor for test-species diversity was applied to all three benchmark types for a chemical contingent upon whether the test-species diversity requirement was met. A chemical was considered to have met the species requirement if test-species reported in ECOTOX included, at a minimum, the following: three unique species of fish, three unique species of invertebrates, and one plant species. Using the test species information from ECOTOX, all test species were gathered into one of the three aforementioned groups. Although most test species fit into these categories well, those that did not were assigned to the species groups thought to be most suitable. Amphibian species were included within the “fish” species category, and species of algae were included within the “plant” species category for our purposes. Because a chemical that has been tested upon a variety of species is presumably better characterized than one not meeting the species requirement, compounds meeting the test species requirement earned an ${AF}_{species}$ of 1. However, if test species requirements were not met for a compound, an ${AF}_{species}$ of 2 was assigned, similar to Hull et al. (2015).

Finally, chemical persistence in an aquatic environment was the last aspect considered in the assignment of AFs in the benchmark development process. Persistence AFs were not applied to Acute Effect benchmarks under the assumption that these higher-degree effects would be elicited even by chemicals that are not persistent. Chemicals with an aquatic half-life shorter than eight weeks were not considered to be persistent whereas those with a half-life of eight weeks or longer were considered persistent (Hull et al. 2015). The aquatic persistence of each chemical was estimated using the BIOWIN^TM^ 3 Ultimate Survey Model within Estimation Programs Interface (EPI) Suite^TM^ (U.S. Environmental Protection Agency 2012). By inputting the CAS number for each detected compound and loading the physical properties, BIOWIN^TM^ 3 was used to gather a raw persistence output value. Then, using the BIOWIN^TM^ 3 persistence value, biodegradation half-lives were estimated as described in Aronson et al. (2006). Chemical compounds with half-lives of less than eight weeks earned an ${AF}_{persistence}$ of 1 whereas chemicals with half-lives of 8 weeks or greater received an ${AF}_{persistence}$ of 5 (Hull et al. 2015).

In summary, using these three considerations (endpoint code, species requirement, and chemical persistence) for AFs, an overall AF was computed by multiplying ${AF}_{endpoint}\times{AF}_{species}\times{AF}_{persistence}$. Overall AF values ranged from 10 to 200. For each chemical the ECOTOX endpoint with the minimum concentration for each Benchmark type was divided by the overall AF calculated for the benchmark. This process resulted in a maximum of three benchmarks for each chemical (depending on ecotoxicity data availability). Of these three possible benchmarks, the lowest (most conservative) benchmark was used for computing toxicity quotient (TQ) values. The most conservative ECOTOX benchmarks were evaluated to ensure the relevance and accuracy of the ECOTOX endpoints from which they were derived. In some cases, the minimum benchmark endpoints were misrepresentative; as a result, they were adjusted or removed. These adjustments are recorded in Table SI-10.

## Hazard quotient comparisons

Considering the EAR threshold of 10^-3^ and the TQ threshold of 0.1 used for evaluation in the present study, the ratio (minimum ACC)/(minimum ECOTOX benchmark) would be 100 if these were representative of similar measures and provided the same level of protection. In the current data sets, ToxCast and ECOTOX information were both available for 25 chemicals. The ratio (minimum ACC)/(minimum ECOTOX benchmark) had a median value of 249 but varied widely among these chemicals with a minimum of 3.09 and a maximum of 6.81 x 10^6^. However, given the differences in the types of assays used to generate underlying data for developing these benchmarks, potentially significant differences are expected. Using an EAR threshold of 10^-3^ and a TQ threshold of 0.1 was part of the conservative approach used in this evaluation in order to provide an additional factor of safety due to uncertainty in the derived benchmark values and the assumption that the maximum stream concentrations were not observed in this study (with only two to four samples collected per site).

# Figures

*
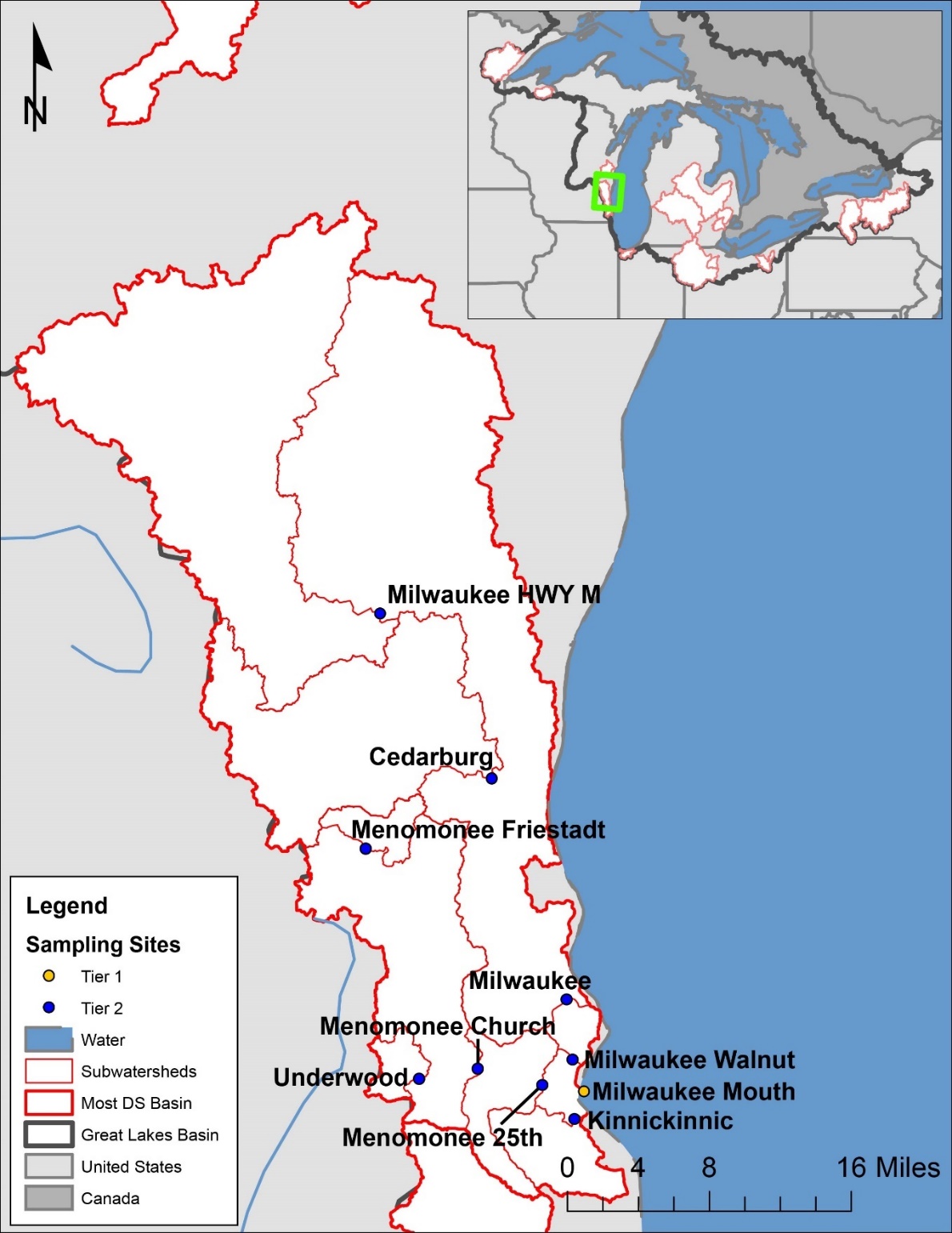
*

Figure SI-1*. Larger-scale map of the Milwaukee River watershed in the United States. Sampling sites are labeled with short names, and their full names can be found in Table SI-1. The small-scale inset map in the upper-righthand corner includes an outline of the large-scale map extent in green.*

*
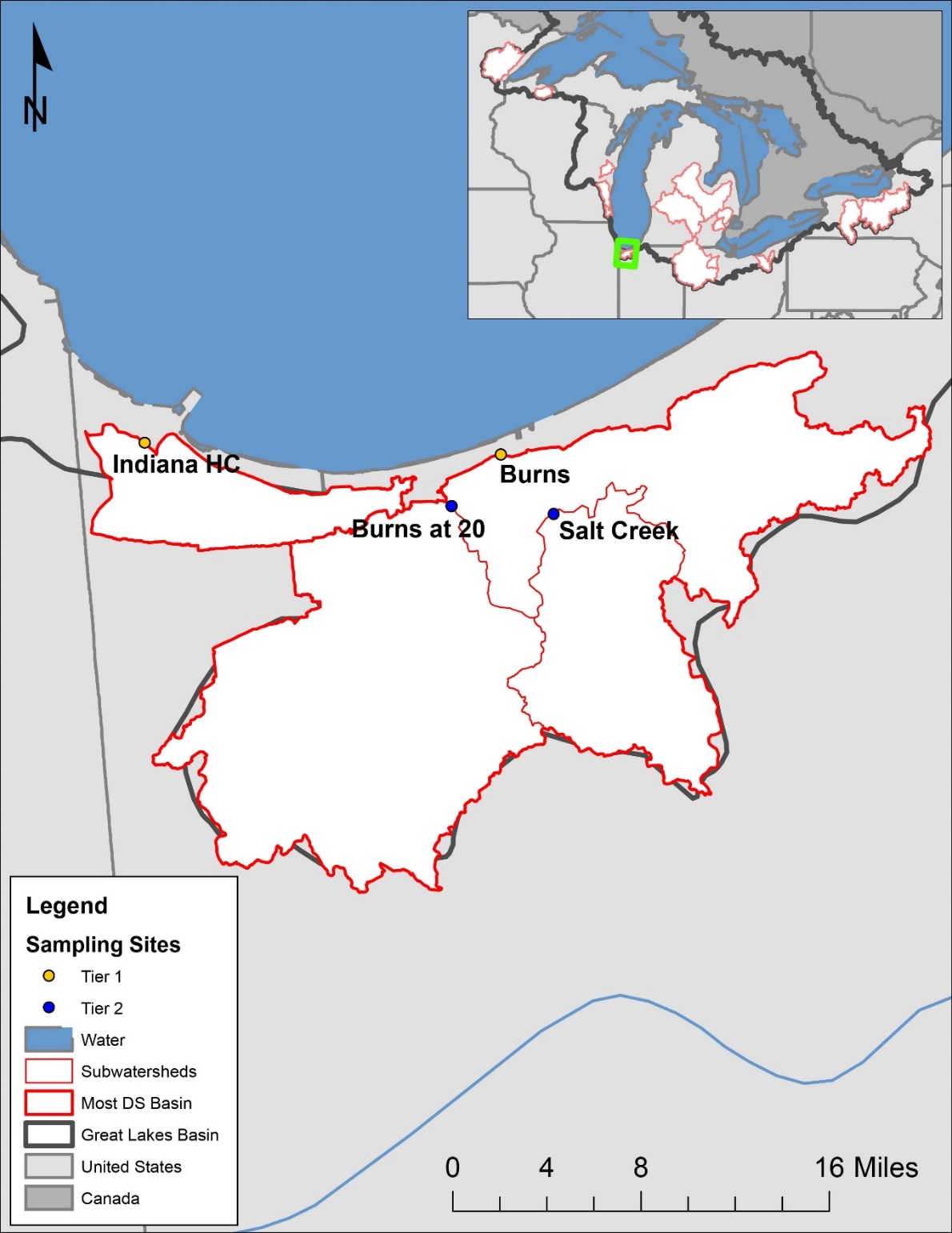
*

Figure SI-2*. Larger-scale map of the Burns River watershed in the United States. Sampling sites are labeled with short names, and their full names can be found in Table SI-1. The small-scale inset map in the upper-righthand corner includes an outline of the large-scale map extent in green.*

*
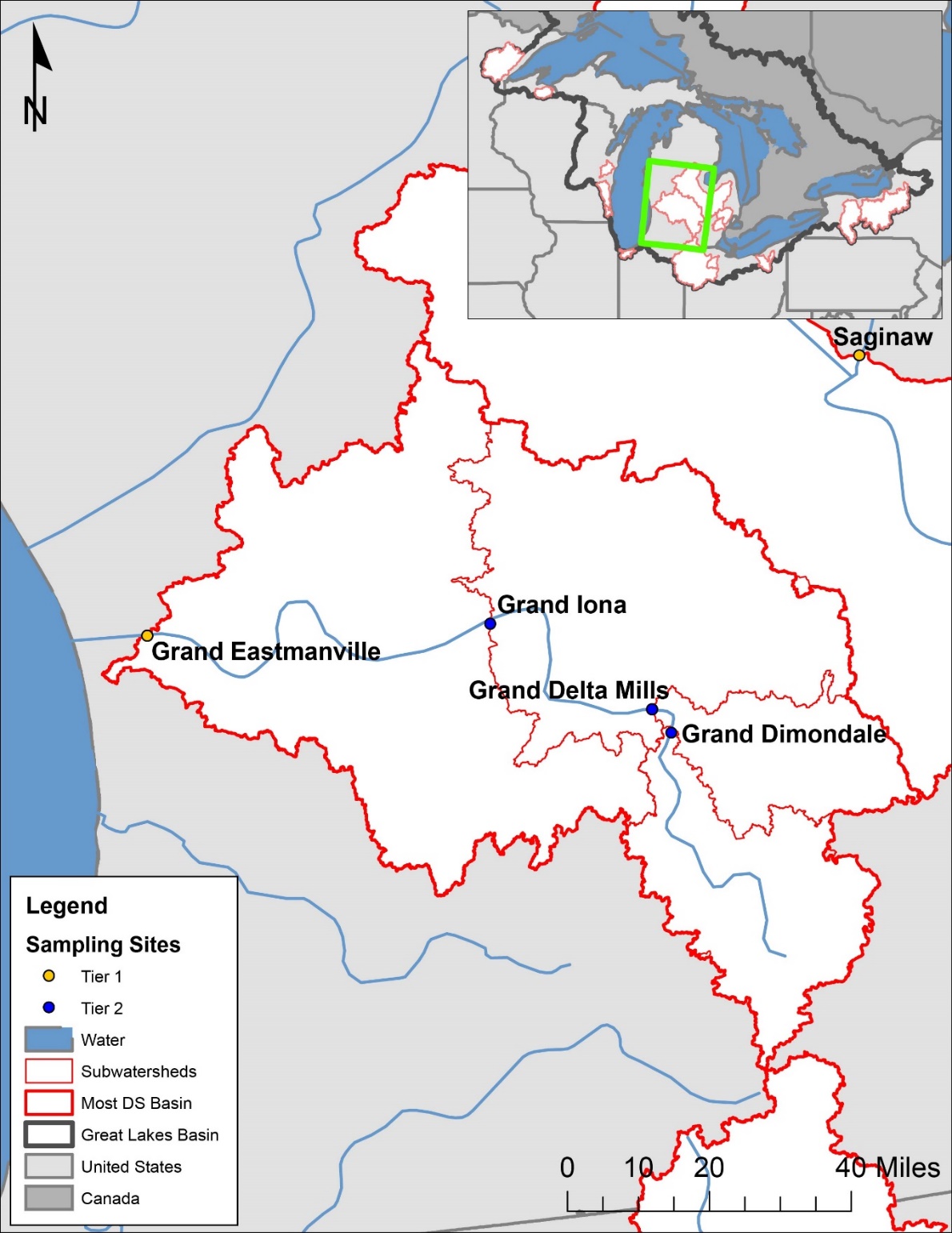
*

*Figure SI-3. Larger-scale map of the Grand River watershed in the United States. Sampling sites are labeled with short names, and their full names can be found in Table SI-1. The small-scale inset map in the upper-righthand corner includes an outline of the large-scale map extent in green.*

*
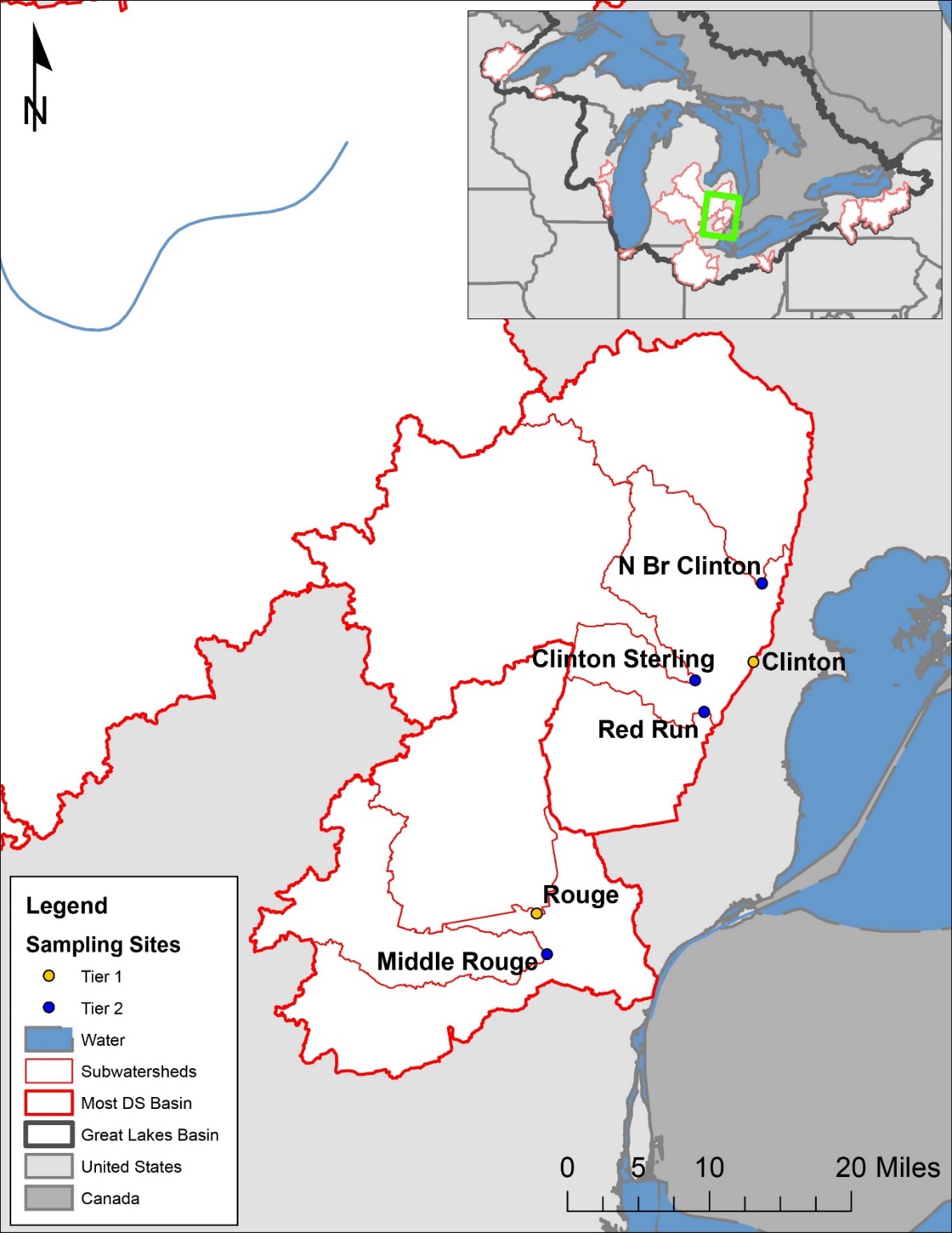
*

Figure SI-4*. Larger-scale map of the Clinton River and Rouge River watersheds in the United States. Sampling sites are labeled with short names, and their full names can be found in Table SI-1. The small-scale inset map in the upper-righthand corner includes an outline of the large-scale map extent in green.*

*
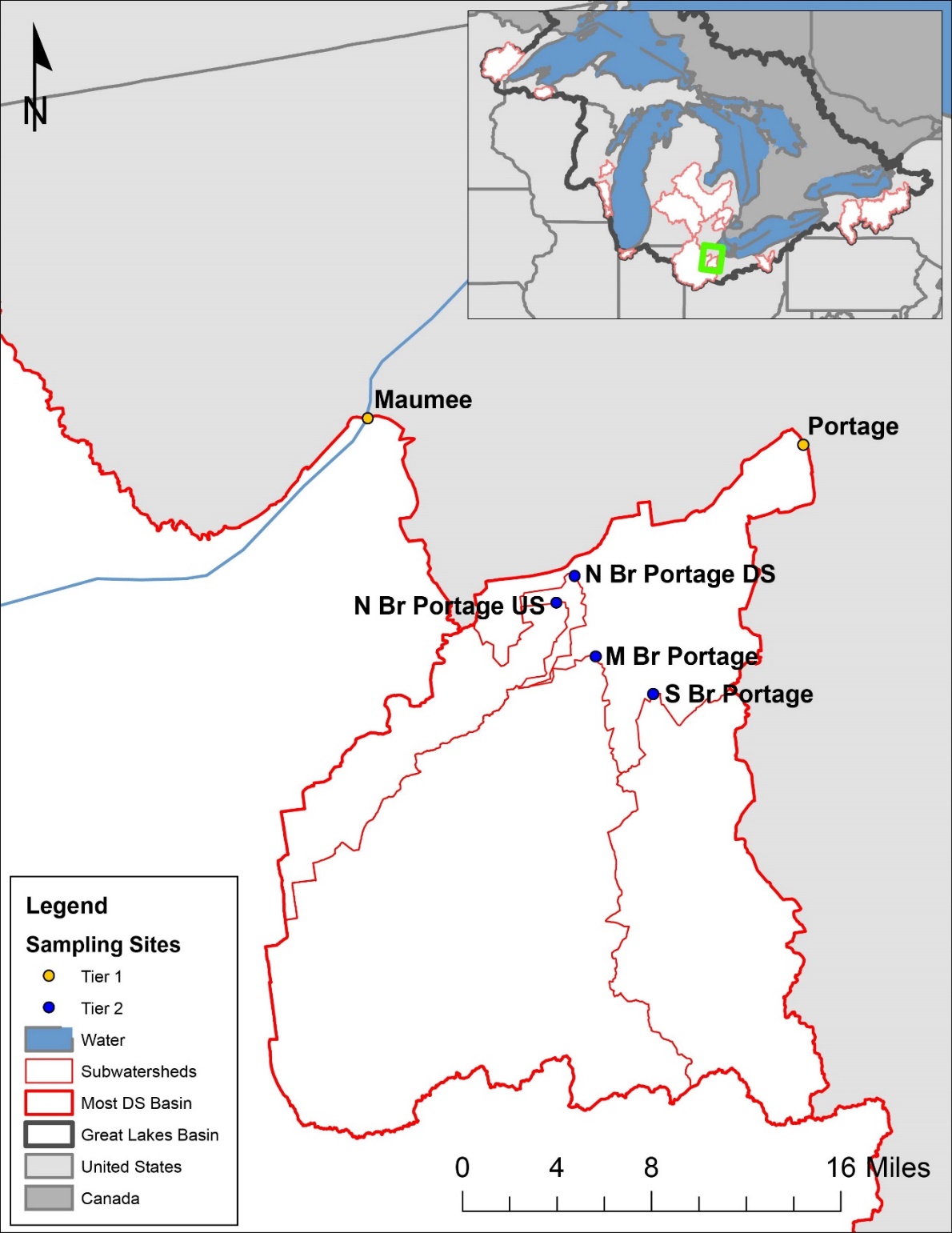
*

Figure SI-5*. Larger-scale map of the Portage River watershed in the United States. Sampling sites are labeled with short names, and their full names can be found in Table SI-1. The small-scale inset map in the upper-righthand corner includes an outline of the large-scale map extent in green.*

*
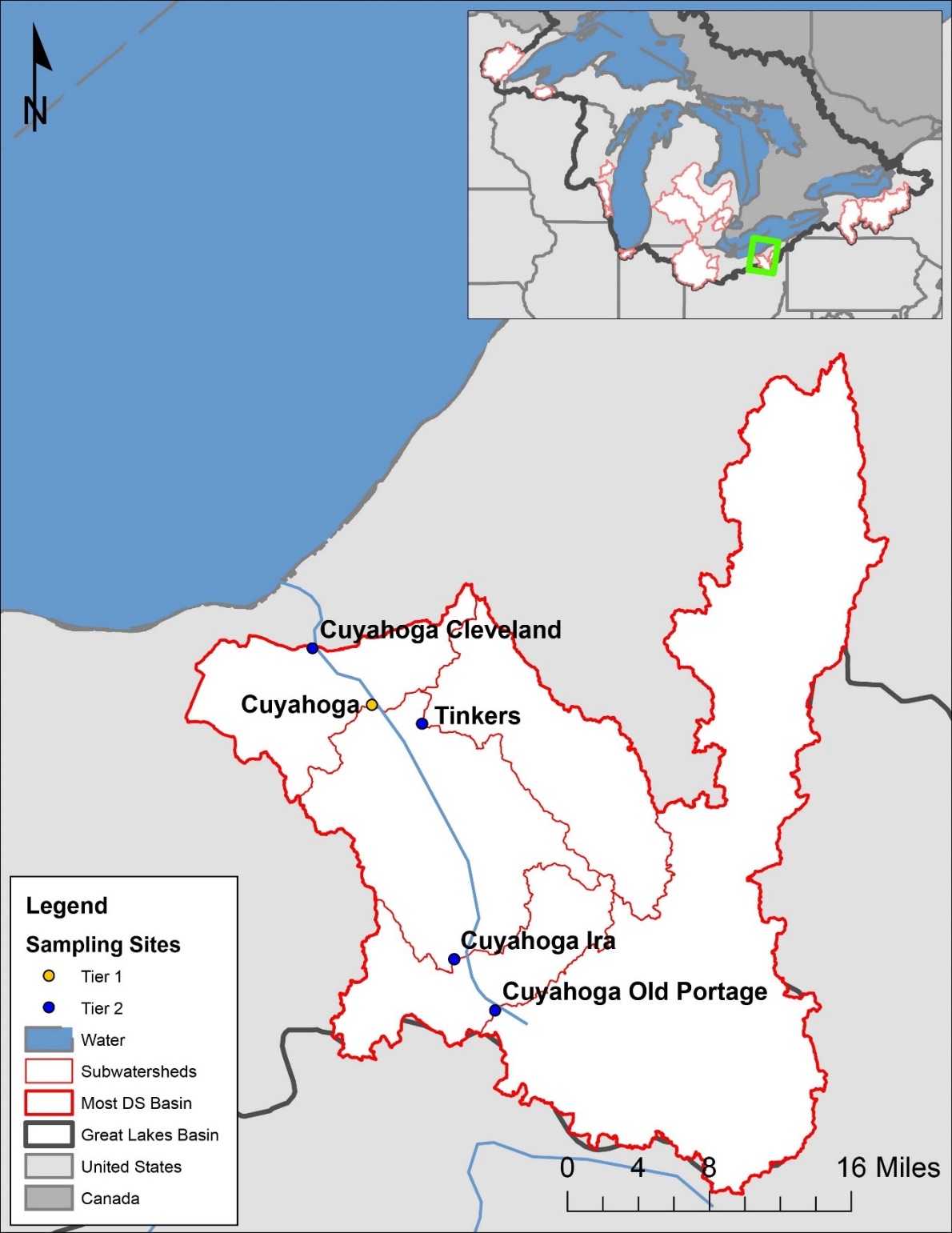
*

Figure SI-6*. Larger-scale map of the Cuyahoga River watershed in the United States. Sampling sites are labeled with short names, and their full names can be found in Table SI-1. The small-scale inset map in the upper-righthand corner includes an outline of the large-scale map extent in green.*

*
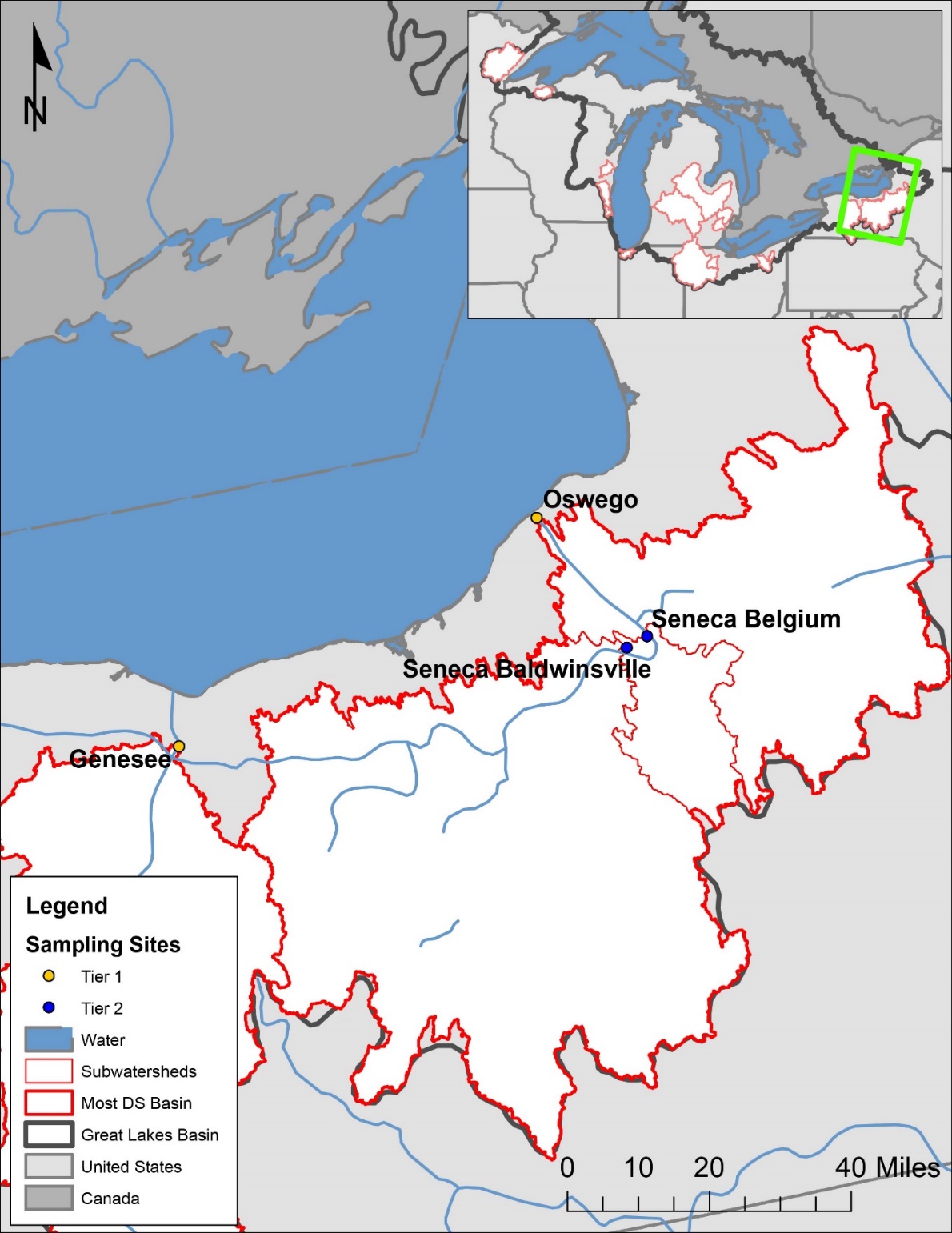
*

Figure SI-7*. Larger-scale map of the Oswego River watershed in the United States. Sampling sites are labeled with short names, and their full names can be found in Table SI-1. The small-scale inset map in the upper-righthand corner includes an outline of the large-scale map extent in green.*

*
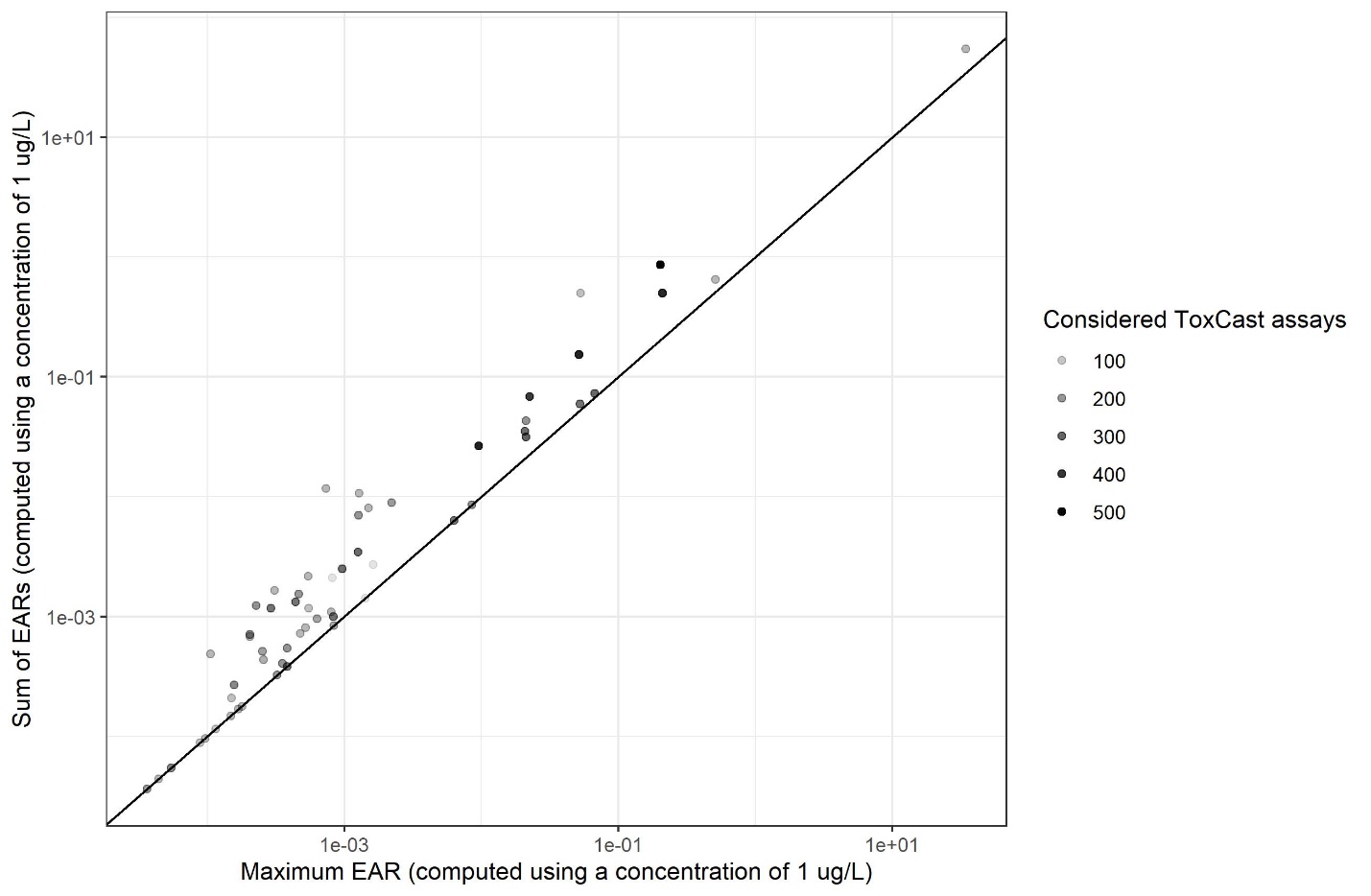
*

Figure SI-8. *Comparison of the maximum Exposure-Activity Ratio (EAR) values (computed from minimum Activity Concentration at Cutoff values) and the sum of EARs (computed from all relevant Activity Concentration at Cutoff values) for chemicals detected in Great Lakes tributary samples (October 2017-September 2018). The shade of each point is part of a gradient representing the number of assays considered for computing the EARs of each chemical. For the purposes of this comparison, EARs were computed using a constant, nominal concentration of 1 µg/L for all chemicals.*


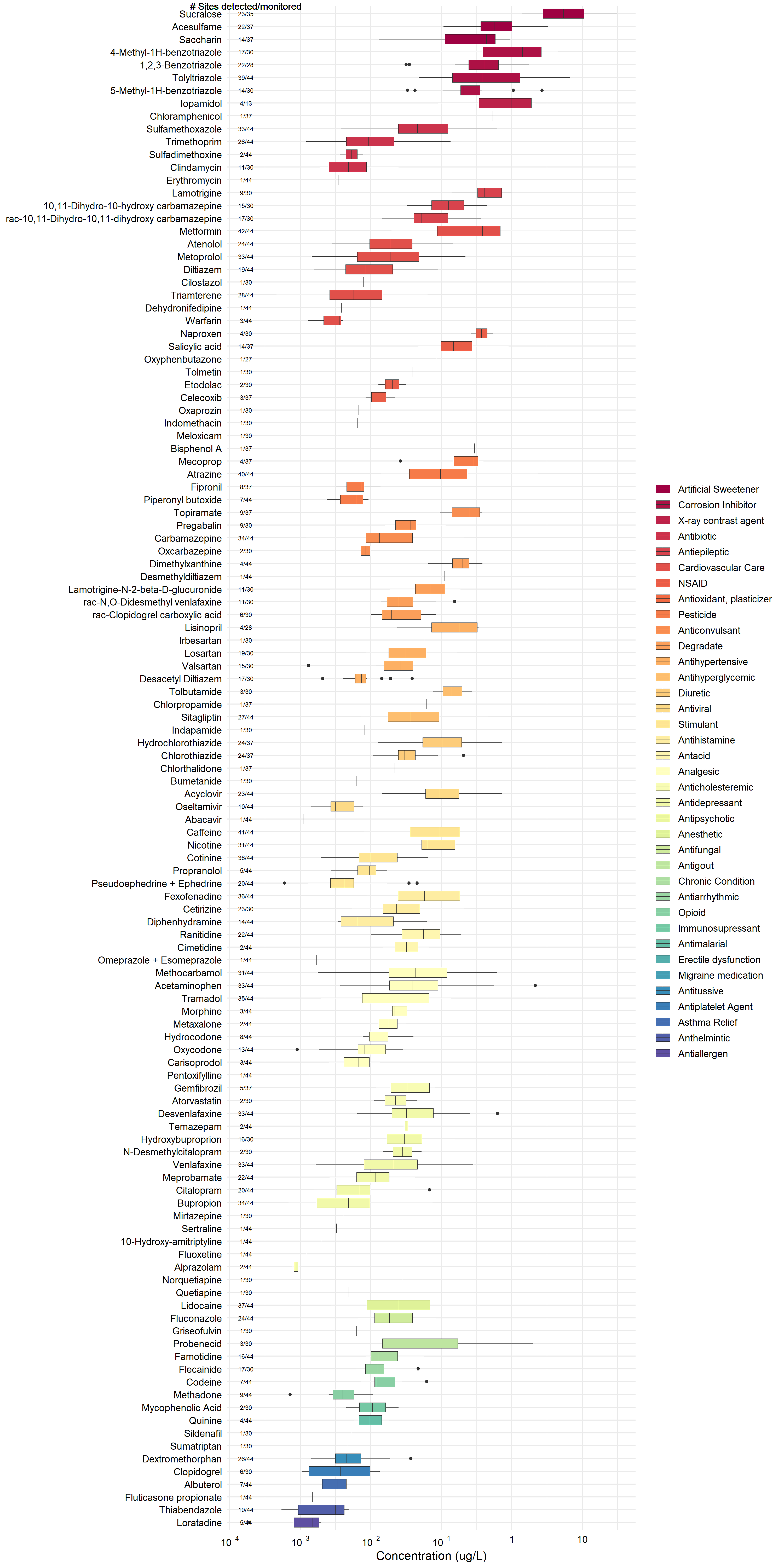


Figure SI-9*. Observed concentrations of all chemicals detected in 44 Great Lakes tributary sampling sites, October 2017-September 2018. Chemicals are in descending order according to median concentration and are grouped by chemical class.* *Boxes depict the first through third quartiles; dark line, median; whiskers, data within 1.5 times the interquartile range; points, outliers.*

*
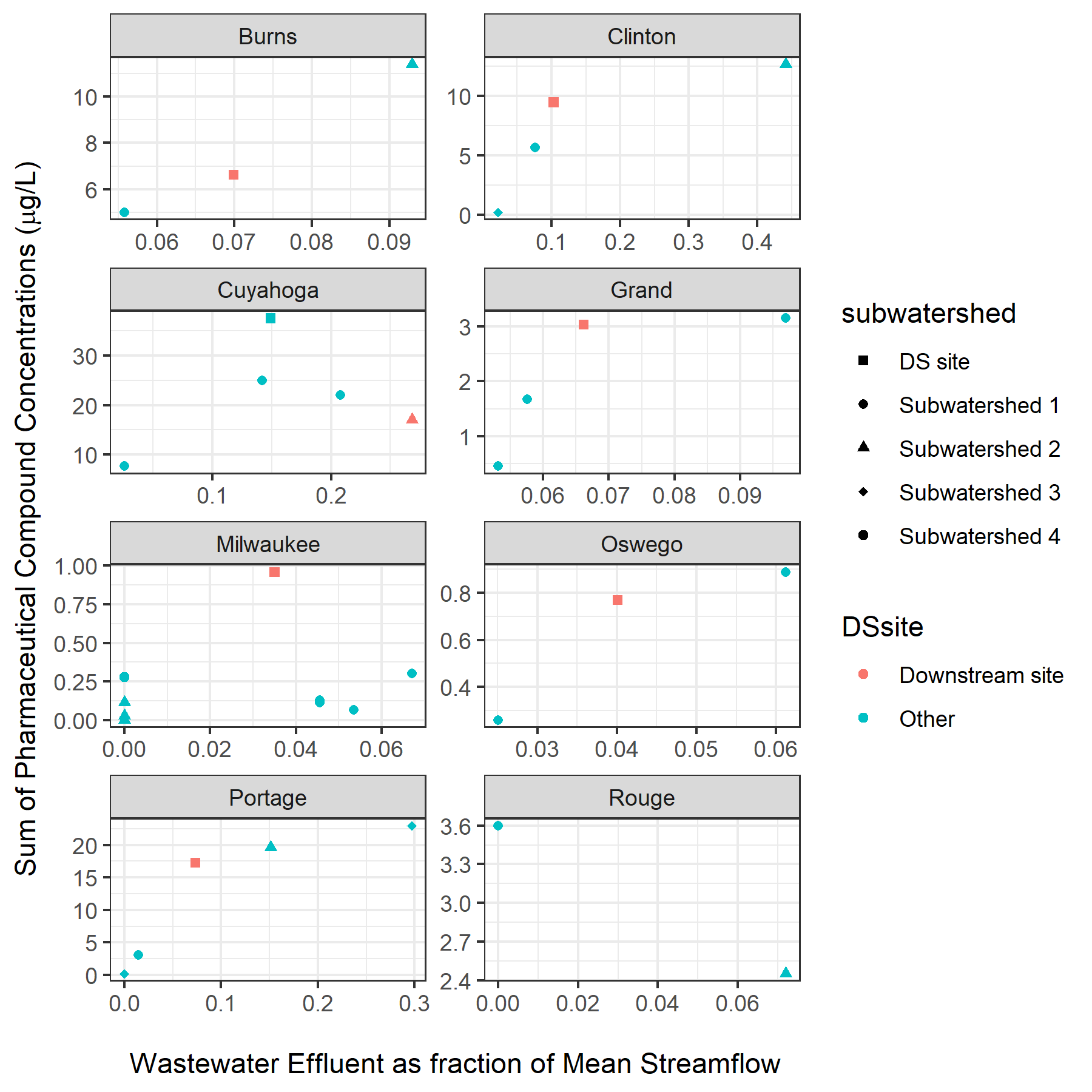
*

Figure SI-10*. Relationship between relative contributions of wastewater treatment plants to streamflow and maximum sum of sampled pharmaceutical and personal care product compound concentrations observed in watersheds with multiple sites. Colors indicate most downstream site and upstream sites. Shapes represent subwatersheds within each watershed that combine to contribute the most downstream site (DS site). River Rouge has two subwatersheds without a downstream site. Samples were collected in Great Lakes tributary sites, October 2017-September 2018.*


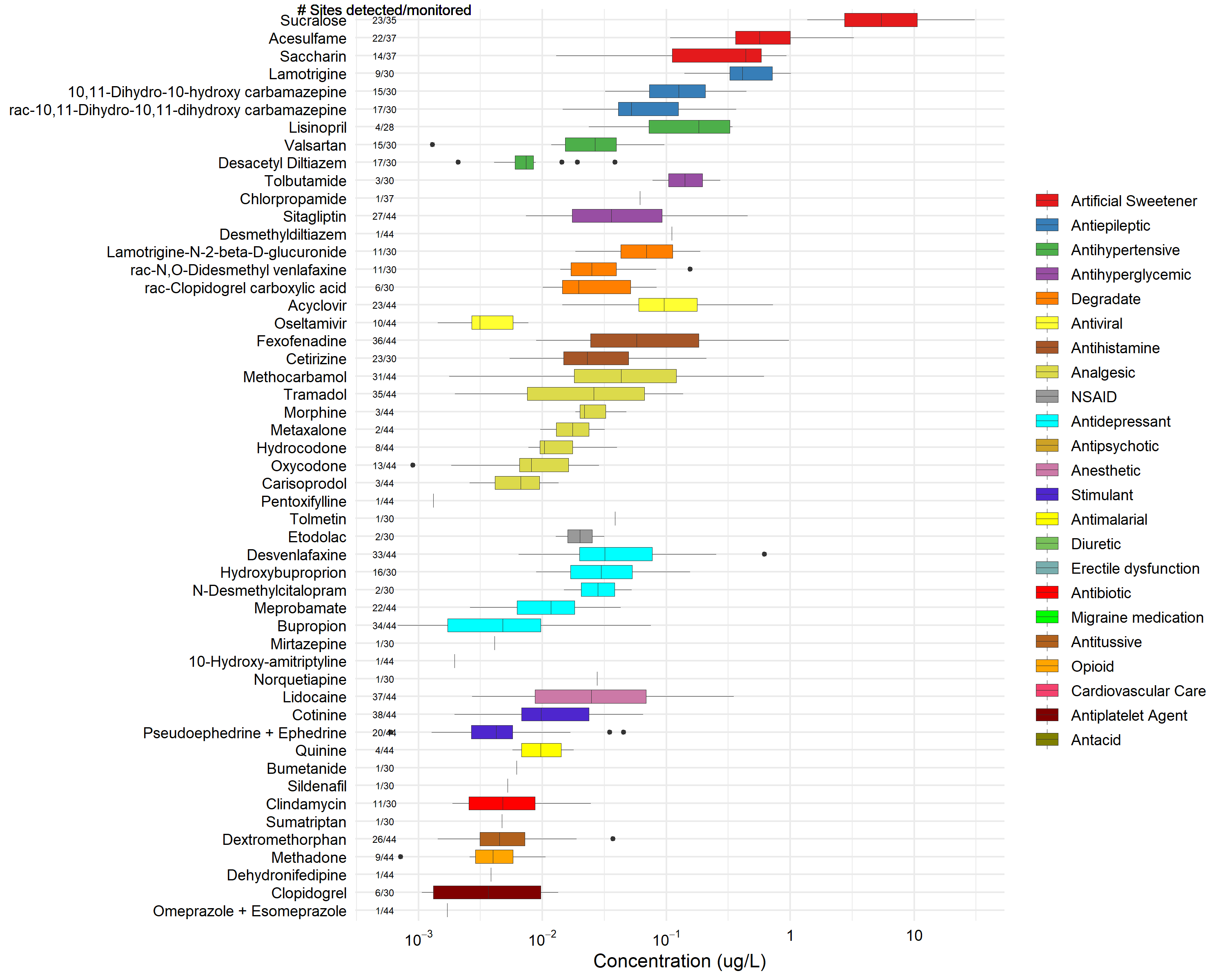


Figure SI-11*. Observed concentrations of chemicals detected in 44 Great Lakes tributary sampling sites, October 2017-September 2018*, *that were not represented by an alternative benchmark, but which were detected at more than 50% of sites monitored for their presence and with a median concentration greater than 0.1 µg/L. Chemicals are in descending order according to median concentration and are grouped by chemical class.* *Boxes depict the first through third quartiles; dark line, median; whiskers, data within 1.5 times the interquartile range; points, outliers.*


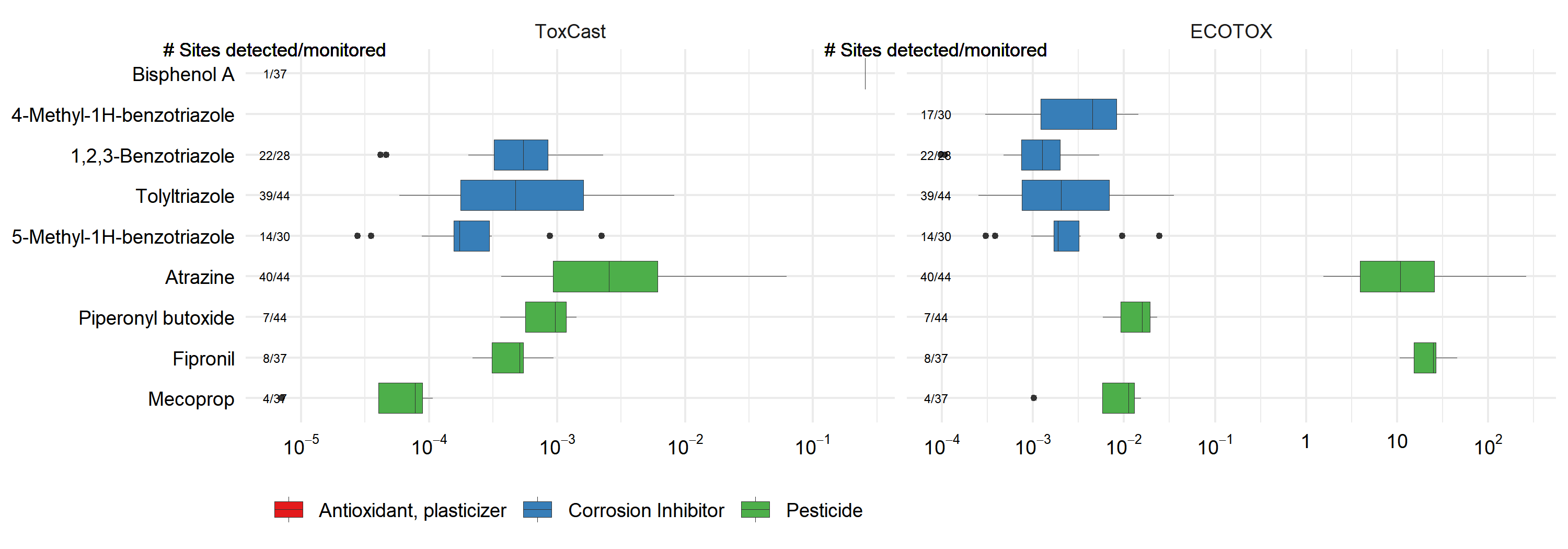


Figure SI-12*. ToxCast exposure-activity ratios, EAR_Chem_, (left) and ECOTOX-derived toxicity quotients, TQ, (right) for non-pharmaceutical chemicals detected in the 44 Great Lakes tributary sampling sites in this study, October 2017-September 2018. Compounds are grouped by chemical class in descending order according to median EAR. In cases where only one screening value was available, the other was intentionally left blank. Chemicals without either screening value were not included. The EAR_Chem_ threshold of concern is EAR_Chem_ > 10^-3^. The TQ threshold of concern is TQ > 10^-1^. Boxes depict the first through third quartiles; dark line, median; whiskers, data within 1.5 times the interquartile range; points, outliers.*

*
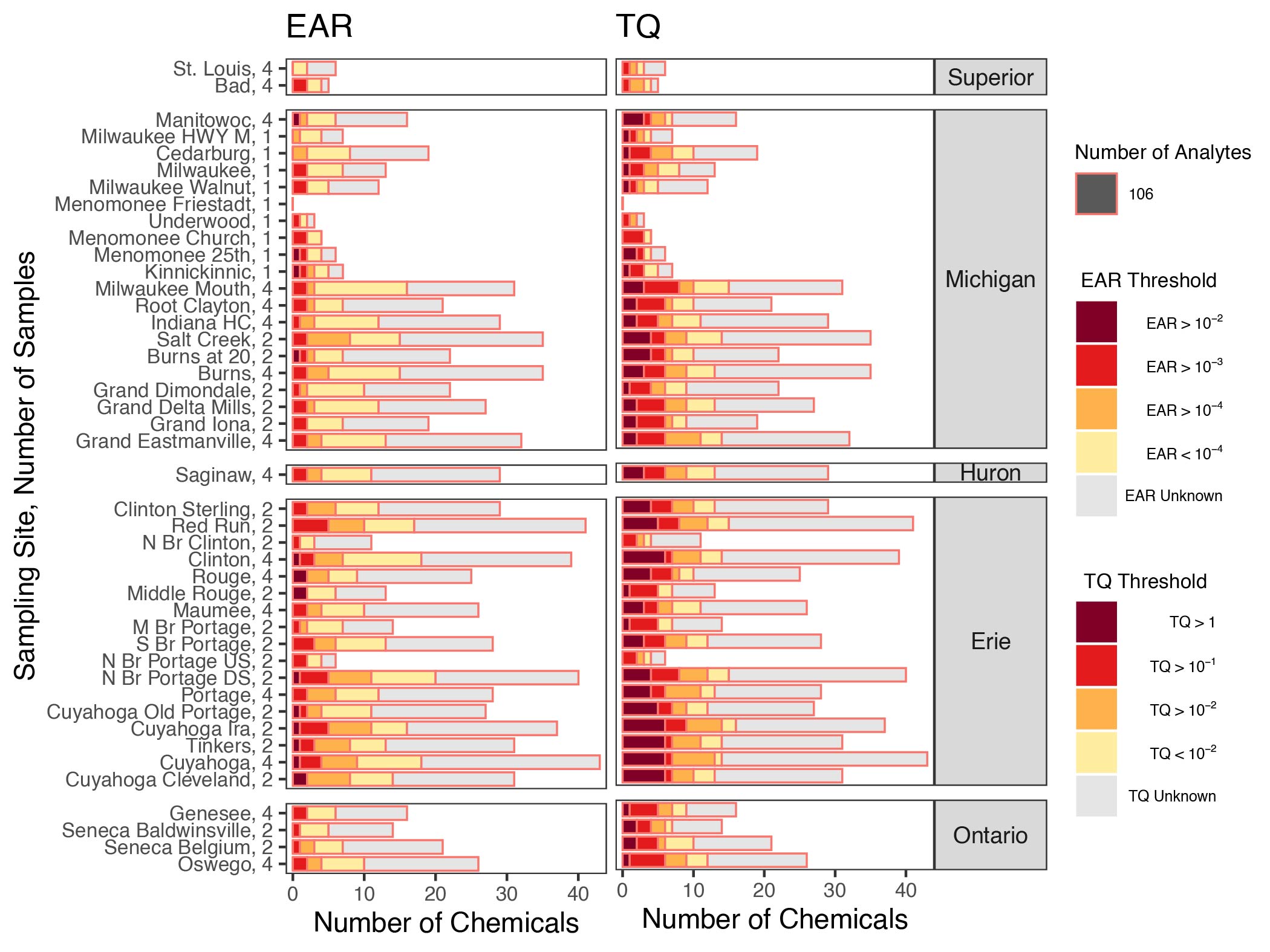
*

Figure SI-13*. Method 1 pharmaceutical compound detections and exceedances of incremental thresholds of concern for ToxCast Exposure-Activity Ratios (left), and ECOTOX-derived Toxicity Quotients (right) organized by sampling site. The number of pharmaceuticals detected at each site are represented by the combination of stacked bars (e.g., 39 pharmaceuticals were detected at Clinton). The border color of the bar indicates that 106 pharmaceuticals were monitored at each site. The number of samples collected from each of the 44 Great Lakes tributary sampling sites, October 2017-September 2018, is listed following the site name.*

*
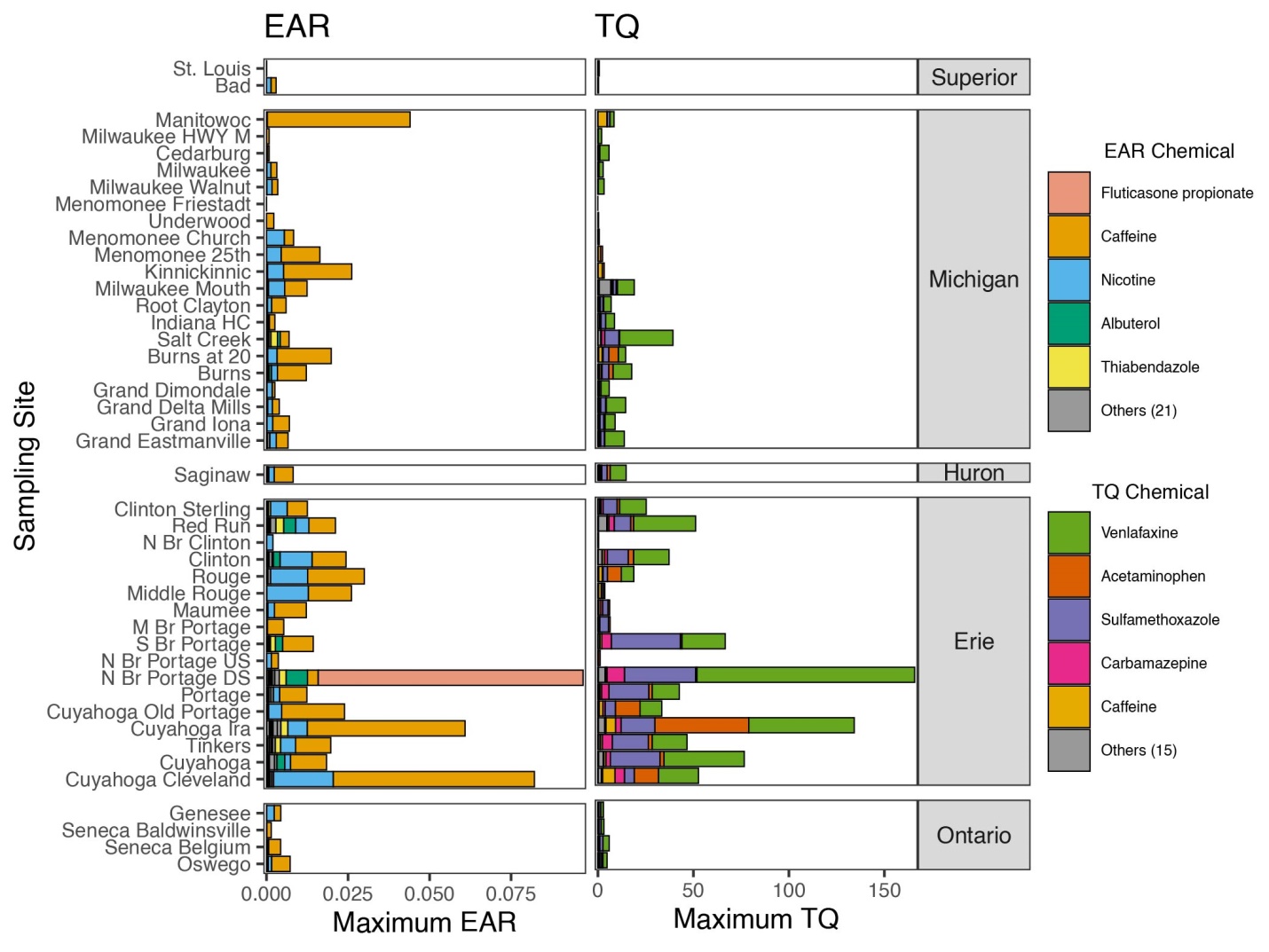
*

Figure SI-14*. Method 1 pharmaceutical compound hazard quotients by site in the Great Lakes Basin from water samples collected in 44 Great Lakes tributary sampling sites, October 2017-September 2018: Sum of maximum ToxCast Exposure-Activity Ratio values for all ToxCast assays (left), and maximum ECOTOX-derived Toxicity Quotients for each pharmaceutical (right). The number of additional pharmaceuticals represented by the respective alternative benchmark is provided in parentheses next to ‘Others’ in the key.*

# References:

Applegate VC, Howell JH, Hall AE, Smith MA. 1957. Toxicity of 4,346 chemicals to larval lampreys and fishes. Special Scientific Report - Fisheries Report No.: 207. http://pubs.er.usgs.gov/publication/70171148.

Aronson D, Boethling R, Howard P, Stiteler W. 2006. Estimating biodegradation half-lives for use in chemical screening. Chemosphere. 63(11):1953–1960. doi:10.1016/j.chemosphere.2005.09.044.

ASTM International. 2016. ASTM D7510 - 10 Standard practice for performing detection and quantitation estimation and data assessment utilizing DQCALC software, based on ASTM practices D6091 and D6512 of Committee D19 on Water. https://www.astm.org/Standards/D7510.htm.

Baldwin AK, Corsi SR, De Cicco LA, Lenaker PL, Lutz MA, Sullivan DJ, Richards KD. 2016. Organic contaminants in Great Lakes tributaries: Prevalence and potential aquatic toxicity. Sci Total Environ. 554–555:42–52. doi:10.1016/j.scitotenv.2016.02.137.

Blackwell BR, Ankley GT, Corsi SR, DeCicco LA, Houck KA, Judson RS, Li S, Martin MT, Murphy E, Schroeder AL, et al. 2017. An “EAR” on Environmental Surveillance and Monitoring: A Case Study on the Use of Exposure–Activity Ratios (EARs) to Prioritize Sites, Chemicals, and Bioactivities of Concern in Great Lakes Waters. Environ Sci Technol. 51(15):8713–8724. doi:10.1021/acs.est.7b01613.

Brain RA, Johnson DJ, Richards SM, Hanson ML, Sanderson H, Lam MW, Young C, Mabury SA, Sibley PK, Solomon KR. 2004. Microcosm evaluation of the effects of an eight pharmaceutical mixture to the aquatic macrophytes Lemna gibba and Myriophyllum sibiricum. Aquat Toxicol. 70(1):23–40. doi:10.1016/j.aquatox.2004.06.011.

Calleja MC, Persoone G, Geladi P. 1994. Comparative acute toxicity of the first 50 Multicentre Evaluation of In Vitro Cytotoxicity chemicals to aquatic non-vertebrates. Arch Environ Contam Toxicol. 26(1):69–78. doi:10.1007/BF00212796.

Childress CJ, Foreman WT, Connor BF, Maloney TJ. 1999. New reporting procedures based on long-term method detection levels and some considerations for interpretations of water-quality data provided by the U.S. Geological Survey National Water Quality Laboratory. U.S. Geological Survey Open-File Report Report No.: 99–193. https://pubs.er.usgs.gov/publication/ofr99193.

Contardo-Jara V, Lorenz C, Pflugmacher S, Nützmann G, Kloas W, Wiegand C. 2011. Exposure to human pharmaceuticals Carbamazepine, Ibuprofen and Bezafibrate causes molecular effects in Dreissena polymorpha. Aquat Toxicol. 105(3–4):428–437. doi:10.1016/j.aquatox.2011.07.017.

Corsi SR, De Cicco LA, Villeneuve DL, Blackwell BR, Fay KA, Ankley GT, Baldwin AK. 2019. Prioritizing chemicals of ecological concern in Great Lakes tributaries using high-throughput screening data and adverse outcome pathways. Sci Total Environ. 686:995–1009. doi:10.1016/j.scitotenv.2019.05.457.

Dawson MA, Renfro JL. 1993. Interaction of structurally similar pesticides with organic anion transport by primary cultures of winter flounder renal proximal tubule. J Pharmacol Exp Ther. 266(2):673.

DeCicco LA, Corsi SR, Villeneuve DL, Blackwell BR, Ankley GT. 2018. toxEval: Evaluation of measured concentration data using the ToxCast high-throughput screening database or a user-defined set of concentration benchmarks. R Package version 1.0.0. https://code.usgs.gov/water/toxEval, doi:10.5066/P906UQ5I.

Divo AA, Geary TG, Jensen JB. 1985. Oxygen- and time-dependent effects of antibiotics and selected mitochondrial inhibitors on Plasmodium falciparum in culture. Antimicrob Agents Chemother. 27(1):21–27. doi:10.1128/AAC.27.1.21.

Eisentraeger A, Dott W, Klein J, Hahn S. 2003. Comparative studies on algal toxicity testing using fluorometric microplate and Erlenmeyer flask growth-inhibition assays. Ecotoxicol Environ Saf. 54(3):346–354. doi:10.1016/S0147-6513(02)00099-4.

European Commission. 2002. European Commission Decision (2002/657/EC) of 12 August 2002 concerning the performance of analytical methods and the interpretation of results: Official Journal of the European Communities Legislation.

Fleming WJ, Ailstock MS, Momot JJ. 1995. Net photosynthesis and respiration of sago pondweed (Potamogeton pectinatus) exposed to herbicides. In: Jane S. Hughes, Gregory R. Biddinger, Eugene Mones, editors. Environmental Toxicology and Risk Assessment: Third Volume. Philadelphia, Pa.: American Society for Testing and Materials. (American Society for Testing and Materials, editor. ). p. 303–317. http://pubs.er.usgs.gov/publication/5210527.

Furlong ET, Noriega MC, Kanagy CJ, Kanagy LK, Coffey LJ, Burkhardt MR. 2014. Determination of human-use pharmaceuticals in filtered water by direct aqueous injection–high-performance liquid chromatography/tandem mass spectrometry.

Gaudet-Hull AM, Rayburn JR, Bantle JA, Burton DT, Turley SD, Dawson DA, Dumont JN, Finch RA, Maurice MA, Fort DJ, et al. 1994. Fetax interlaboratory validation study: Phase II testing. Environ Toxicol Chem. 13(10):1629–1637. doi:10.1002/etc.5620131012.

Hayasaka Daisuke, Korenaga T, Sánchez-Bayo F, Goka K. 2012. Differences in ecological impacts of systemic insecticides with different physicochemical properties on biocenosis of experimental paddy fields. Ecotoxicology. 21(1):191–201. doi:10.1007/s10646-011-0778-y.

Hayasaka D., Korenaga T, Suzuki K, Saito F, Sánchez-Bayo F, Goka K. 2012. Cumulative ecological impacts of two successive annual treatments of imidacloprid and fipronil on aquatic communities of paddy mesocosms. Ecotoxicol Environ Saf. 80:355–362. doi:10.1016/j.ecoenv.2012.04.004.

Hull RN, Kleywegt S, Schroeder J. 2015. Risk-based screening of selected contaminants in the Great Lakes Basin. J Gt Lakes Res. 41(1):238–245. doi:10.1016/j.jglr.2014.11.013.

Jin S, Homer C, Yang L, Danielson P, Dewitz J, Li C, Zhu Z, Xian G, Howard D. 2019. Overall Methodology Design for the United States National Land Cover Database 2016 Products. Remote Sens. 11(24):2971. doi:10.3390/rs11242971.

Kish P. 2004. Effects of Roundup, Glean, Aatrex, and Their Active Ingredients (Glyphosate, Chlorsulfuron, And Atrazine) on Periphyton Communities Studied by Using Matlock Periphytometer and Bottle Tests. Oklahoma State University. https://shareok.org/bitstream/handle/11244/44099/Thesis-2004D-K61e.pdf?sequence=1&isAllowed=y.

Kloas W, Lutz I, Springer T, Krueger H, Wolf J, Holden L, Hosmer A. 2009. Does Atrazine Influence Larval Development and Sexual Differentiation in Xenopus laevis? Toxicol Sci. 107(2):376–384. doi:10.1093/toxsci/kfn232.

Laitta MT. 2016. Waste Water Treatment Plants in the Great Lakes. [accessed 2021 Mar 16]. https://usgs.maps.arcgis.com/home/item.html?id=e4a5b201232944c2a671eb96621274dd.

López-Doval JC, Kukkonen JVK, Rodrigo P, Muñoz I. 2012. Effects of indomethacin and propranolol on Chironomus riparius and Physella (Costatella) acuta. Ecotoxicol Environ Saf. 78:110–115. doi:10.1016/j.ecoenv.2011.11.004.

Maupin MA, Ivahnenko T. 2011. Nutrient Loadings to Streams of the Continental United States from Municipal and Industrial Effluent1: Nutrient Loadings to Streams of the Continental United States From Municipal and Industrial Effluent. JAWRA J Am Water Resour Assoc. 47(5):950–964. doi:10.1111/j.1752-1688.2011.00576.x.

Mimeault C, Woodhouse AJ, Miao X-S, Metcalfe CD, Moon TW, Trudeau VL. 2005. The human lipid regulator, gemfibrozil bioconcentrates and reduces testosterone in the goldfish, Carassius auratus. Aquat Toxicol. 73(1):44–54. doi:10.1016/j.aquatox.2005.01.009.

Müller H-G. 1982. Sensitivity of Daphnia magna straus against eight chemotherapeutic agents and two dyes. Bull Environ Contam Toxicol. 28(1):1–2. doi:10.1007/BF01608403.

Nentwig G. 2008. Another Example of Effects of Pharmaceuticals on Aquatic Invertebrates: Fluoxetine and Ciprofloxacin. In: Kümmerer K, editor. Pharmaceuticals in the Environment. Berlin, Heidelberg: Springer Berlin Heidelberg. p. 205–222. [accessed 2022 Feb 3]. http://link.springer.com/10.1007/978-3-540-74664-5_13.

Nichols JW, Du B, Berninger JP, Connors KA, Chambliss CK, Erickson RJ, Hoffman AD, Brooks BW. 2015. Observed and modeled effects of pH on bioconcentration of diphenhydramine, a weakly basic pharmaceutical, in fathead minnows: pH influence on BCF of diphenhydramine in fathead minnows. Environ Toxicol Chem. 34(6):1425–1435. doi:10.1002/etc.2948.

Nickell LG, Finlay AC. 1954. Growth Modifiers, Antibiotics and Their Effects on Plant Growth. J Agric Food Chem. 2(4):178–182. doi:10.1021/jf60024a003.

Olsén KH, Ask K, Olsén H, Porsch-Hällström I, Hallgren S. 2014. Effects of the SSRI citalopram on behaviours connected to stress and reproduction in Endler guppy, Poecilia wingei. Aquat Toxicol. 148:113–121. doi:10.1016/j.aquatox.2013.12.032.

Rocco L, Frenzilli G, Fusco D, Peluso C, Stingo V. 2010. Evaluation of zebrafish DNA integrity after exposure to pharmacological agents present in aquatic environments. Ecotoxicol Environ Saf. 73(7):1530–1536. doi:10.1016/j.ecoenv.2010.07.032.

Rocco L, Frenzilli G, Zito G, Archimandritis A, Peluso C, Stingo V. 2012. Genotoxic effects in fish induced by pharmacological agents present in the sewage of some Italian water-treatment plants. Environ Toxicol. 27(1):18–25. doi:10.1002/tox.20607.

Schoenfuss HL, Furlong ET, Phillips PJ, Scott T-M, Kolpin DW, Cetkovic-Cvrlje M, Lesteberg KE, Rearick DC. 2016. Complex mixtures, complex responses: Assessing pharmaceutical mixtures using field and laboratory approaches: Biological effects of complex pharmaceutical mixtures. Environ Toxicol Chem. 35(4):953–965. doi:10.1002/etc.3147.

Shridhar S, Hassan K, Sullivan DJ, Vasta GR, Fernández Robledo JA. 2013. Quantitative assessment of the proliferation of the protozoan parasite Perkinsus marinus using a bioluminescence assay for ATP content. Int J Parasitol Drugs Drug Resist. 3:85–92. doi:10.1016/j.ijpddr.2013.03.001.

Tillitt DE, Papoulias DM, Whyte JJ, Richter CA. 2010. Atrazine reduces reproduction in fathead minnow (Pimephales promelas). Aquat Toxicol. 99(2):149–159. doi:10.1016/j.aquatox.2010.04.011.

Traba HM, Domínguez-Morueco N, Barreno E, Catalá M. 2017. Lichen microalgae are sensitive to environmental concentrations of atrazine. J Environ Sci Health Part B. 52(4):223–228. doi:10.1080/03601234.2016.1270679.

U.S. Census Bureau Geography Division. 2010. 2010 State-based Census Block TIGER/Line Shapefile. Washington, D.C.: U.S. Department of Commerce, U.S. Census Bureau, Geography Division, Geographic Products Branch. [accessed 2022 Jan 26]. https://www.census.gov/geographies/mapping-files/time-series/geo/tiger-line-file.2010.html.

U.S. Department of Agriculture-Natural Resources Conservation Service, U. S. Geological Survey, U.S Environmental Protection Agency. 2009. The Watershed Boundary Dataset (WBD). Fort Worth, Texas: U.S. Department of Agriculture, Natural Resources Conservation Service, National Cartography and Geospatial Center. [accessed 2012 Jan 25]. http://datagateway.nrcs.usda.gov.

U.S. Environmental Protection Agency. 2005. Guidelines Establishing Test Procedures for the Analysis of Pollutants Appendix B - Definition and Procedure for the Determination of the Method Detection Limit. Protection of Environment Report No.: 40 CFR Part 136. https://www.govinfo.gov/app/details/CFR-2020-title40-vol25/CFR-2020-title40-vol25-part136-appB.

U.S. Environmental Protection Agency. 2012. Estimation Programs Interface Suite. Washington, DC; United States. https://www.epa.gov/tsca-screening-tools/epi-suitetm-estimation-program-interface.

U.S. Environmental Protection Agency. 2017. ToxCast & Tox21 Summary Files from invitrodb_v3.2. US EPA. [accessed 2020 May 5]. https://www.epa.gov/chemical-research/exploring-toxcast-data-downloadable-data.

U.S. Environmental Protection Agency, U.S. Geological Survey. 2012. National Hydrography Dataset Plus - NHDPlus (Edition 2.10). Washington, D.C.: U.S. Environmental Protection Agency. http://www.horizon-systems.com/nhdplus/NHDPlusV2_home.php.

U.S. Geological Survey. 2002. Chapter A5. Processing of water samples. [accessed 2021 Mar 29]. https://pubs.er.usgs.gov/publication/twri09A5.

U.S. Geological Survey. 2004. Chapter A3. Cleaning of equipment for water sampling. [accessed 2021 Mar 29]. http://pubs.er.usgs.gov/publication/twri09A3.

U.S. Geological Survey. 2006. Chapter A4. Collection of water samples. [accessed 2021 Mar 29]. http://pubs.er.usgs.gov/publication/twri09A4.

Wickham J, Stehman SV, Sorenson DG, Gass L, Dewitz JA. 2021. Thematic accuracy assessment of the NLCD 2016 land cover for the conterminous United States. Remote Sens Environ. 257:112357. doi:10.1016/j.rse.2021.112357.

Williams T, Foreman WT, Decess J, Reed-Parker C, Stevenson DL. 2015. Changes to National Water Quality Laboratory (NWQL) procedures used to establish and verify laboratory detection and reporting limits. U.S. Geological Survey National Water Quality Laboratory Technical Memorandum 15.02. https://nwql.usgs.gov/tech_memos/nwql.2015-02.pdf.

Wood RJ, Mitrovic SM, Lim RP, Kefford BJ. 2016. How benthic diatoms within natural communities respond to eight common herbicides with different modes of action. Sci Total Environ. 557–558:636–643. doi:10.1016/j.scitotenv.2016.03.142.

Yang L, Jin S, Danielson P, Homer C, Gass L, Bender SM, Case A, Costello C, Dewitz J, Fry J, et al. 2018. A new generation of the United States National Land Cover Database: Requirements, research priorities, design, and implementation strategies. ISPRS J Photogramm Remote Sens. 146:108–123. doi:10.1016/j.isprsjprs.2018.09.006.
